# Oncometabolite fumarate facilitates PD-L1 expression and immune evasion in clear cell renal cell carcinoma

**DOI:** 10.1038/s41419-025-07752-4

**Published:** 2025-06-03

**Authors:** Yi Gao, Shiyin Fan, Xue Sun, Jiaxi Li, Yue Dai, Hongchen Li, Haijie Ma, Yanping Xu, Lei Lv

**Affiliations:** 1 Ministry of Education Key Laboratory of Metabolism and Molecular Medicine, Department of Biochemistry and Molecular Biology, School of Basic Medical Sciences, Fudan University, Shanghai, China; 2 Tongji Hospital, Shanghai Key Laboratory of Signaling and Disease Research, Frontier Science Center for Stem Cell Research, School of Life Sciences and Technology, Tongji University, Shanghai, China; 3 Cellular and Molecular Biology Laboratory, Affiliated Zhoushan Hospital of Wenzhou Medical University, Zhoushan, Zhejiang China

**Keywords:** Tumour immunology, Cancer metabolism, Cancer microenvironment

## Abstract

Clear cell renal cell carcinoma (ccRCC) is the most common subtype of renal cell carcinoma (RCC), with a rising incidence worldwide. However, the mechanisms by which ccRCC evades immune surveillance remain incompletely understood. Our findings indicate that fumarate hydratase (FH) expression is significantly downregulated in ccRCC, resulting in fumarate accumulation, which is correlated with a poor prognosis in ccRCC patients. RNA sequencing analysis suggests that dimethyl fumarate (DMF), an FDA-approved fumarate analogue, may impact tumor immunity. Our further investigation reveals that both DMF and the FH inhibitor (FHIN1) can promote immune evasion in ccRCC by upregulating PD-L1. Pre-treatment of tumor cells with DMF notably inhibits the cytotoxic effect of T cells. Mechanistically, fumarate induces PD-L1 expression through succination of HIF-1α at C800, facilitating its interaction with importin α3, p300, and PKM2, which promotes HIF-1α nuclear localization and transcriptional activity. Moreover, combining DMF with PD-L1 blockade therapy significantly enhances the efficacy of immunotherapy and prolongs the survival of tumor-bearing mice. Taken together, our study elucidates a mechanism by which FH downregulation promotes immune evasion through the fumarate-HIF-1α/p300/PKM2-PD-L1 axis, providing a novel target, drug, and strategy to improve immunotherapy for ccRCC.

## Introduction

Clear cell renal cell carcinoma (ccRCC) originates from the epithelium of the renal tubules in the renal parenchyma and is a common malignant tumor of the urinary system [1]. It accounts for ~75% of renal cell carcinoma (RCC) cases [2] and is characterized by distinct genetic abnormalities, most notably the loss of function of the von Hippel-Lindau (VHL) tumor suppressor gene [3, 4]. ccRCC also exhibits a high degree of invasiveness and metastasis, leading to a generally poorer prognosis compared to other subtypes [5]. Over the past few decades, the treatment strategies for ccRCC have encompassed surgery, chemotherapy, radiotherapy, cytokine therapy, and immune checkpoint blockade (ICB) [6]. Since the FDA approval of nivolumab in 2015, various immune checkpoint inhibitors (ICIs) have been widely applied in the treatment of ccRCC and have become crucial therapeutic approaches [7]. However, ICB is not effective for all patients, and its therapeutic efficacy is influenced by multiple factors such as individual differences, tumor staging, and pathological types [8]. Overall, the treatment of ccRCC still faces significant challenges, and exploring efficient systemic therapies and enhancing the efficacy of immunotherapy remain key objectives of current research [9, 10].

Programmed death-ligand 1 (PD-L1), also known as B7-H1, is a type I transmembrane protein with a molecular weight of 33 kilodaltons (kDa) [11]. PD-L1 is composed of an extracellular domain, a transmembrane domain, and an intracellular domain. The extracellular domain features an N-terminal immunoglobulin-like domain (IgV) that binds to programmed cell death 1 (PD-1) [12], a critical interaction for immune system regulation. The most well-known ICIs are anti-PD-1 and anti-PD-L1 monoclonal antibodies. These inhibitors block the PD-1/PD-L1 interaction, thereby activating the T-cell receptor signaling pathway and enhancing tumor immunity [13, 14]. Clinical trials in ccRCC patients have shown that those with high PD-L1 expression have significantly longer median survival compared to those with low PD-L1 expression [15]. This suggests that PD-L1 expression levels could serve as an important biomarker for predicting the efficacy of ICI treatment in ccRCC [15, 16]. It is important to note that PD-L1 expression in ccRCC tumors varies widely [9, 17]. Consequently, the therapeutic effect of anti-PD-L1 antibodies is less effective in patients with low PD-L1 expression [18, 19]. This variability presents a significant clinical challenge and highlights the need for more effective therapeutic options. While boosting immune function is essential for tumor elimination, it is still unclear whether dysregulation of fumarate hydratase (FH) expression or function in ccRCC affects tumor immune evasion or PD-L1 expression.

Fumarate plays a multifaceted role in oncogenic signaling pathways, with its accumulation in cancer cells typically resulting from loss-of-function mutations or low expression of the tumor suppressor enzyme FH, a crucial component of the tricarboxylic acid (TCA) cycle. Dimethyl fumarate (DMF), a derivative of the TCA intermediate fumarate, was first approved by the US Food and Drug Administration (FDA) for the treatment of multiple sclerosis and psoriasis due to its immunomodulatory and antioxidative properties [20, 21]. DMF can covalently modify cysteine residues of Kelch-like ECH-associated protein 1 (KEAP1) through succination, leading to the activation of nuclear factor erythroid 2-related factor 2 (Nrf2), which generates antioxidant and anti-inflammatory effects [22, 23]. Additionally, DMF inhibits the nuclear entry of NF-κB family members, effectively blocking the NF-κB signaling pathway and significantly reducing the production of pro-inflammatory cytokines [24, 25]. However, the role of DMF in treating ccRCC and its underlying mechanisms remains unclear. Here, we demonstrate for the first time that fumarate can upregulate PD-L1 expression through succination of HIF-1α, thereby promoting immune evasion in ccRCC. Importantly, the combination of DMF administration and anti-PD-L1 antibody significantly enhanced the efficacy of immunotherapy in a mouse model of renal cancer, indicating that DMF is a promising adjuvant for immunotherapy.

## Results

### Fumarate facilitates immune evasion in ccRCC

**Fig. 1:**
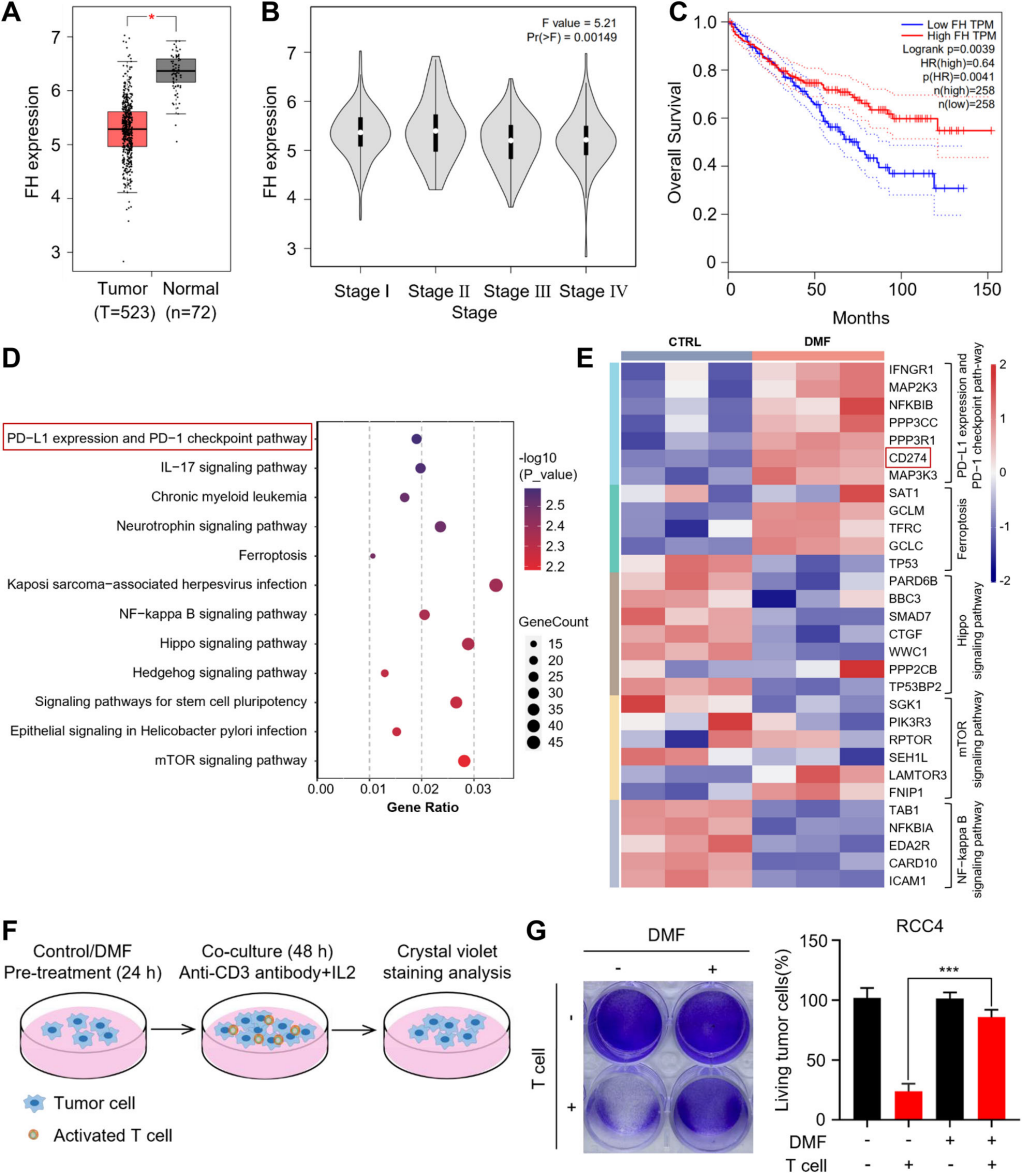
Accumulation in fumarate triggers alterations of immune response in ccRCC. **A** Differences in the expression of FH between ccRCC and normal samples are shown using the GEPIA platform. *P < 0.05. **B** FH expression levels in normal samples and four different tumor stages of ccRCC. *P < 0.05. **C** Kaplan–Meier curves for overall survival (OS) of ccRCC patients with low and high FH expression levels. **D**, **E** RNA-seq analysis was performed on RCC4 cells with and without DMF treatment, using three biologically independent samples per group. All listed genes showed significant differences (*P* < 0.05). **F** Schematic diagram depicting the determination of the effect of DMF on T cell-mediated killing of RCC4 cells. **G** Control and DMF–treated RCC4 cells were cocultured with activated T cell for 48 h and then subjected to crystal violet staining. The quantification was shown on the Right. Data represent mean ± SD, n = 3. ***P < 0.001.

To investigate the role of FH in the development of ccRCC, we analyzed its expression and association with prognosis. Our results indicated that FH is significantly downregulated in ccRCC compared to normal tissues, and its expression level inversely correlates with tumor stages I–IV (Fig. 1A, B). Additionally, patients with lower FH expression tend to have shorter survival periods (Fig. 1C). To elucidate the mechanisms underlying this clinical observation, we performed RNA-seq analysis. Given that low expression of FH leads to significant accumulation of fumarate in ccRCC, we analyzed the gene expression differences in RCC4 cells under DMF treatment. KEGG analysis revealed that DMF regulates multiple cancer-related pathways, including PD-L1 expression and PD-1 checkpoint, ferroptosis, and Hippo signaling pathways (Fig. 1D, E). To confirm whether DMF regulates tumor immunity, we measured the impact of DMF on T cell-mediated tumor cell killing. Activated T cells were co-cultured with RCC4 cells with or without DMF pretreatment (Fig. 1F). The results demonstrated that pretreatment of tumor cells with DMF significantly inhibited the killing effect of T cells (Fig. 1G). Collectively, these findings suggest that fumarate profoundly affects the tumor microenvironment and contributes to the immune evasion of ccRCC, thereby influencing disease progression, treatment response, and overall prognosis.

### Fumarate promotes PD-L1 transcription in ccRCC

**Fig. 2:**
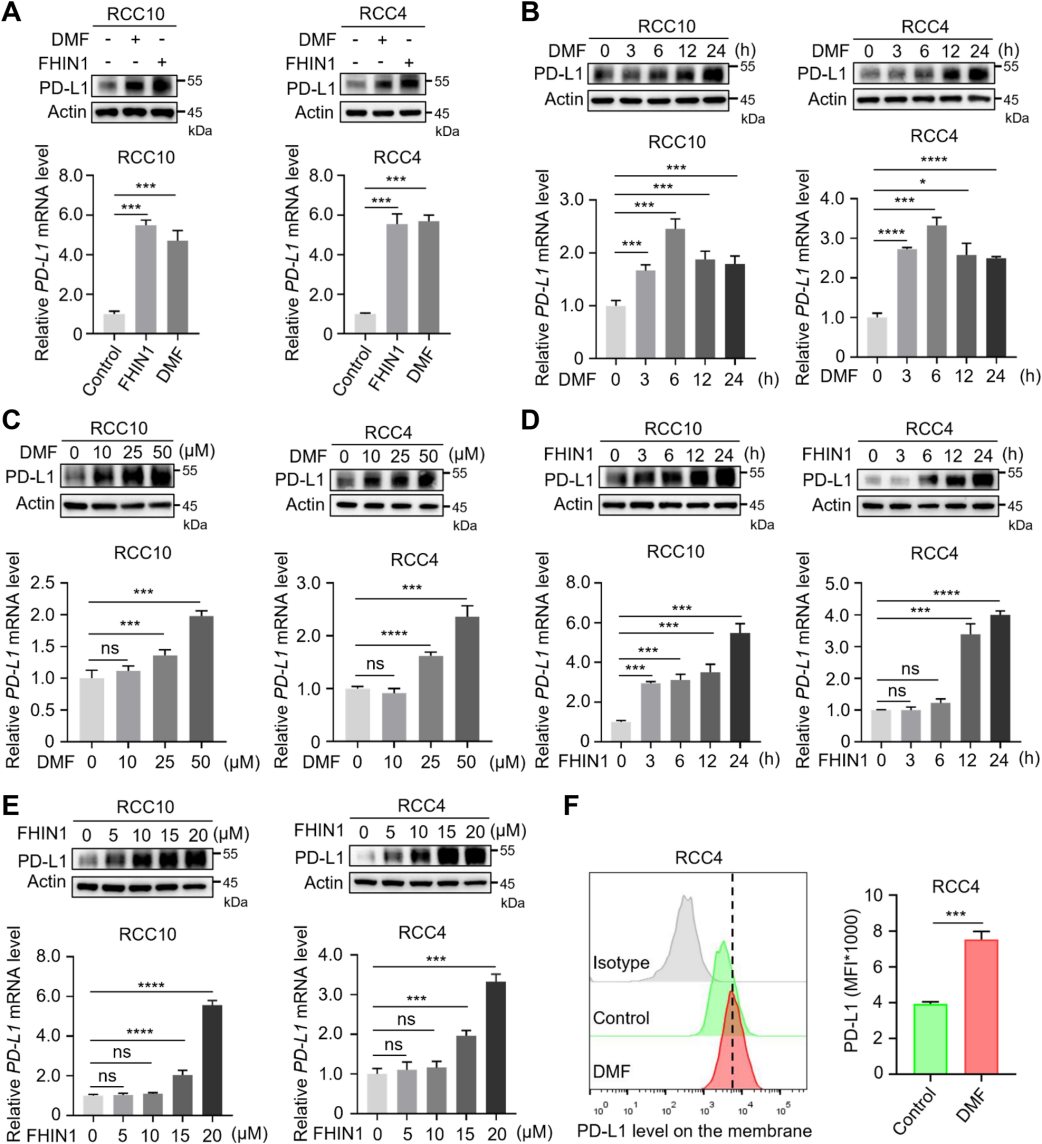
Fumarate upregulates the expression of PD-L1 via transcriptional regulation in ccRCC. **A** Western blot and qRT-PCR analyses of PD-L1 in RCC10 and RCC4 cells with or without DMF (50 μM, 12 h) or FHIN1 (20 μM, 24 h) were performed. ***P < 0.001. **B** Western blot and qRT-PCR analyses of PD-L1 expression in RCC10 and RCC4 cells treated with 50 μM DMF for various durations as indicated. *P < 0.05, ***P < 0.001, ****P < 0.0001. **C** Western blot and qRT-PCR analyses of PD-L1 expression in RCC10 and RCC4 cells treated with increasing concentrations of DMF (0–50 μM) for 12 h. ns nonsignificant, ***P < 0.001, ****P < 0.0001. **D** Western blot and qRT-PCR analyses evaluated PD-L1 expression in RCC10 and RCC4 cells treated with 20 μM FHIN1 at 3 h, 6 h, 12 h, and 24 h. ns, nonsignificant, ***P < 0.001, ****P < 0.0001. **E** Western blot and qRT-PCR analyses were used to assess PD-L1 expression levels in RCC10 and RCC4 cells after treatment with FHIN1 concentrations ranging from 0 to 20 μM for 24 h. ns nonsignificant, ***P < 0.001, ****P < 0.0001. **F** Flow cytometry analysis of membrane PD-L1 expression in RCC4 cells under DMF treatment. Representative histograms and summarized mean fluorescent intensity (MFI) are shown. Values are means ± SD from n = 3 independent experiments. Statistical differences were determined by Student’s *t*-test. ***P < 0.001.

Previous data indicated that fumarate may promote PD-L1 transcription (Fig. 1E). To confirm this, we examined the effects of DMF and the FH inhibitor (FHIN1) [25] on PD-L1 expression in ccRCC cells. The results demonstrated that FHIN1, similar to DMF treatment, upregulated both the protein and mRNA levels of PD-L1 in RCC10 and RCC4 cells (Fig. 2A and Supplementary Fig. 1A, B). Notably, this upregulation was both concentration- and time-dependent (Fig. 2B–E). Furthermore, using two different siRNAs to knock down FH in RCC10 and RCC4 cells (Supplementary Fig. 1C), we found that PD-L1 protein and mRNA levels were also upregulated (Supplementary Fig. 1D). Additionally, treatment with Actinomycin D, which inhibits DNA transcription, suppressed DMF-induced PD-L1 upregulation (Supplementary Fig. 1E). Consistently, DMF treatment did not alter the half-life of PD-L1 (Supplementary Fig. 1F). In addition, we used flow cytometry to detect PD-L1 levels on the cell surface under DMF treatment. The results showed that DMF significantly increased the level of PD-L1 on the cell surface of RCC4 cells (Fig. 2F). In summary, these data suggest that DMF/FHIN1-induced fumarate accumulation promotes PD-L1 transcription in ccRCC.

### Fumarate upregulates PD-L1 expression via HIF-1α

**Fig. 3:**
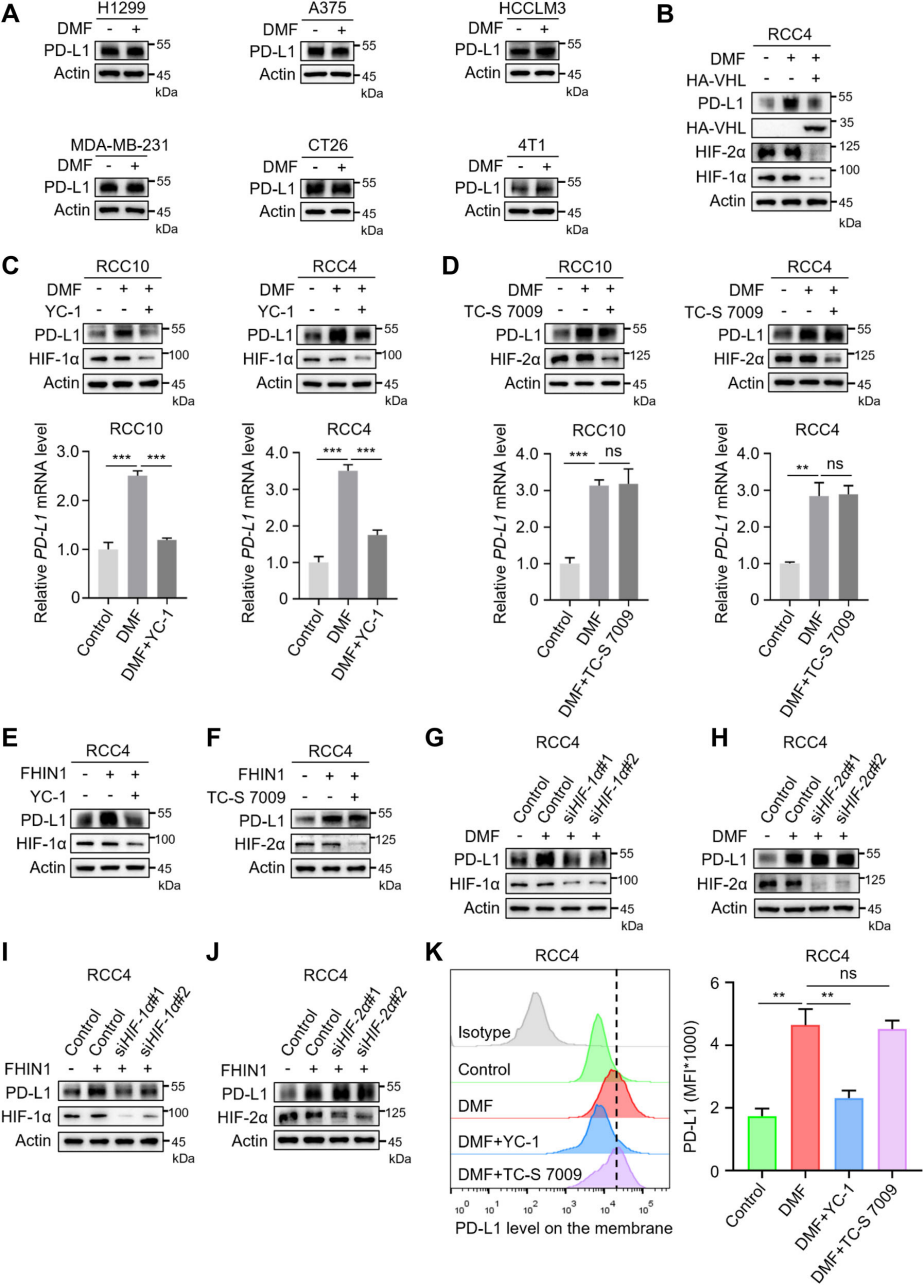
Fumarate facilitates PD-L1 expression via HIF-1α. **A** Western blot analysis was performed to assess the protein levels in four human tumor cell lines (non-small cell lung cancer H1299, melanoma A375, liver cancer HCCLM3, breast cancer MDA-MB-231) and two mouse tumor cell lines (colorectal cancer CT26, breast cancer 4T1) following treatment with 50 μM DMF for 12 h. **B** The expression levels of HIF-1α, HIF-2α, and PD-L1 in RCC4 cells with or without transfection of VHL-expressing plasmids, were examined by Western blot after treatment with DMF (50 μM, 12 h). **C** Western blot and qRT-PCR analyses were performed to assess the PD-L1 levels in RCC10 and RCC4 cells treated with DMF, in the absence and presence of 5 μM YC-1, an inhibitor of HIF-1α. ***P < 0.001. **D** Western blot and qRT-PCR analyses were performed to assess PD-L1 levels in RCC10 and RCC4 cells treated with DMF, with or without 50 μM TC-S 7009, an inhibitor of HIF-2α. ns nonsignificant, **P < 0.01, ***P < 0.001. **E** Western blot analyses were performed to assess the PD-L1 levels in RCC4 cells treated with FHIN1, both in the absence and presence of 5 μM YC-1, which is an inhibitor of HIF-1α. **F** Western blot analyses were performed to assess PD-L1 levels in RCC4 cells treated with FHIN1 with or without 50 μM TC-S 7009, which is an inhibitor of HIF-2α. **G** After transfection with two siRNA fragments designed to knock down HIF-1α expression, RCC4 cells were treated with DMF, followed by the detection of PD-L1 expression levels through western blot. **H** After transfection of RCC4 cells with two HIF-2α-knockdown siRNAs, treatment with DMF was followed by detection of PD-L1 expression using western blot. **I** After transfection with two siRNA fragments designed to knockdown HIF-1α expression, RCC4 cells were treated with FHIN1, followed by the detection of PD-L1 expression levels through western blot. **J** After transfection of RCC4 cells with two HIF-2α-knockdown siRNAs, treatment with FHIN1 was followed by detection of PD-L1 expression using western blot. **K** Following pretreatment with YC-1 or TC-S 7009, the membrane PD-L1 expression in DMF-treated RCC4 cells was analyzed using flow cytometry. Representative histograms and summarized mean fluorescent intensity (MFI) are shown. Values are means ± SD from n = 3 independent experiments. Statistical differences were determined by Student’s *t-*test. ns nonsignificant, **P < 0.01.

We next investigated whether DMF has a similar effect on other tumor cell lines. Four human tumor cell lines (non-small cell lung cancer H1299, melanoma A375, liver cancer HCCLM3, breast cancer MDA-MB-231) and two mouse tumor cell lines (colorectal cancer CT26, breast cancer 4T1) were selected. Surprisingly, DMF had little to no effect on PD-L1 expression in these tumor cells (Fig. 3A and Supplementary Fig. 2A). This led us to explore why DMF specifically upregulates PD-L1 levels in ccRCC. The inactivation of the VHL tumor suppressor gene is a hallmark of ccRCC, occurring in ~90% of patients [26], and leads to the accumulation of HIF-1/2α even under normoxic conditions [27, 28]. Since HIF-1/2α can also promote PD-L1 transcription, we hypothesized that DMF might regulate PD-L1 through HIF-1/2α. To validate the hypothesis, we overexpressed VHL in RCC4 cells and examined the protein levels of HIF-1/2α. Interestingly, VHL-induced degradation of HIF1/2α almost completely abolished the effect of DMF on PD-L1 expression (Fig. 3B). Additionally, HIF-1α inhibitors PX-478 and YC-1 can inhibit DMF/FHIN1-mediated upregulation of PD-L1 in ccRCC cells, whereas the HIF-2α inhibitor TC-S 7009 has no effect (Fig. 3C–F, Supplementary Fig. 2B). To further validate the role of HIF-1/2α in fumarate-induced PD-L1 upregulation, we employed small interfering RNA (siRNA) to knock down HIF-1α expression in RCC4 cells (Supplementary Fig. 2C, D), and the results were consistent with those obtained using HIF-1/2α inhibitors (Fig. 3G–J, Supplementary Fig. 2E, F). Consistently, flow cytometry analysis revealed that HIF-1α inhibitor YC-1, but not the HIF-2α inhibitor TC-S 7009, significantly reduced DMF-induced upregulation of PD-L1 levels on tumor cell surfaces (Fig. 3K). Taken together, these findings suggest that DMF/FHIN1 may upregulate PD-L1 expression through HIF-1α, rather than HIF-2α.

### Fumarate promotes PD-L1 expression through the HIF-1α/p300/PKM2 transcriptional complex

**Fig. 4:**
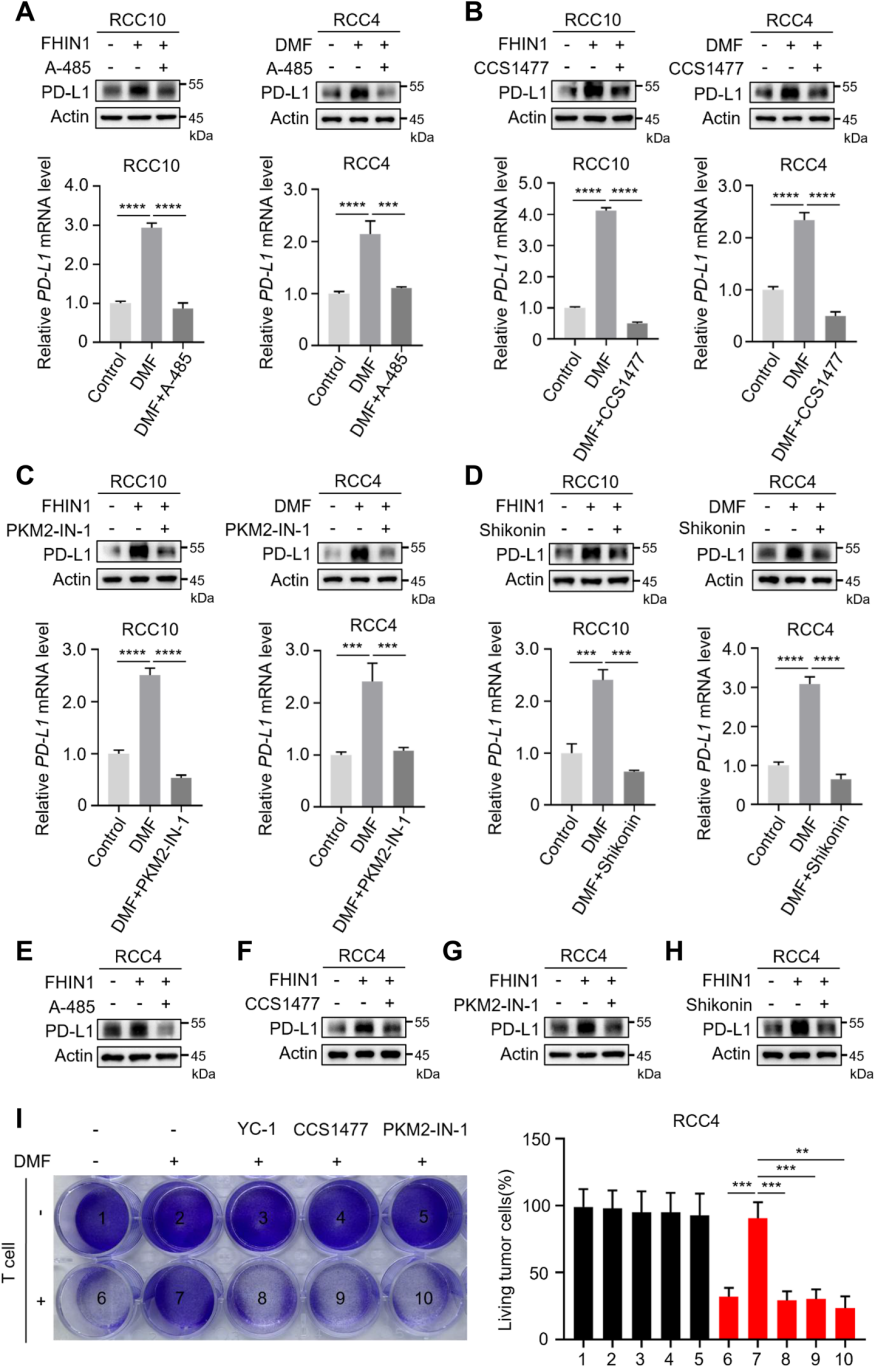
Fumarate-induced PD-L1 expression depends on HIF-1α/p300/PKM2 transcriptional complex. **A**, **B** Western blot and qRT-PCR analyses were performed to evaluate the PD-L1 levels in RCC10 and RCC4 cells treated with DMF/FHIN1, both in the absence and presence of 20 μM A485 or 100 nM CCS1477, an inhibitor of p300. ***P < 0.001, ****P < 0.0001. **C**, **D** Western blot and qRT-PCR analyses were performed to assess PD-L1 levels in RCC10 and RCC4 cells that were treated with DMF/FHIN1, with or without the addition of 0.3 μM PKM2-IN-1 or 1 μM Shikonin, an inhibitor of PKM2. ***P < 0.001, ****P < 0.0001. **E**, **F** Western blot analyses were performed to assess the PD-L1 levels in RCC4 cells treated with FHIN1, both in the absence and presence of 20 μM A485 or 100 nM CCS1477, an inhibitor of p300. **G**, **H** Western blot analyses were performed to assess PD-L1 levels in RCC4 cells that were treated with FHIN1, with or without the addition of 0.3 μM PKM2-IN-1 or 1 μM Shikonin, an inhibitor of PKM2. **I** After pre-treatment with or without YC-1, CCS1477, and PKM2-IN-1, DMF-treated RCC4 cells were cocultured with activated T cells for 48 h, followed by crystal violet staining. The quantification was shown on the Right. Data represent mean ± SD, n = 3. **P < 0.01, ***P < 0.001.

Previous studies have shown that PKM2 can interact directly with HIF-1α, enhancing the transcriptional activation of target genes by promoting HIF-1α binding and the recruitment of p300 to hypoxia-responsive elements [29]. To determine whether PKM2 and p300 are involved in fumarate-induced PD-L1 expression, we used p300 inhibitors A-485 and CCS1477 (Fig. 4A, B, E, F), as well as PKM2 inhibitors PKM2-IN-1 and Shikonin (Fig. 4C, D, G, H). The results demonstrated that these inhibitors significantly suppressed DMF/FHIN1-induced upregulation of PD-L1 in RCC10 and RCC4 cells (Fig. 4A–H). Additionally, we assessed the effects of DMF in the presence of inhibitors for HIF-1α, p300, and PKM2 on T cell-mediated tumor cell killing. We found that these inhibitors eliminated the inhibitory effect of DMF-pretreated tumor cells on T cell killing (Fig. 4I). Collectively, these findings suggest that the upregulation of PD-L1 by fumarate may rely on the HIF-1α/p300/PKM2 transcriptional complex.

### Fumarate facilitates PD-L1 expression by succination and activation of HIF-1α

**Fig. 5:**
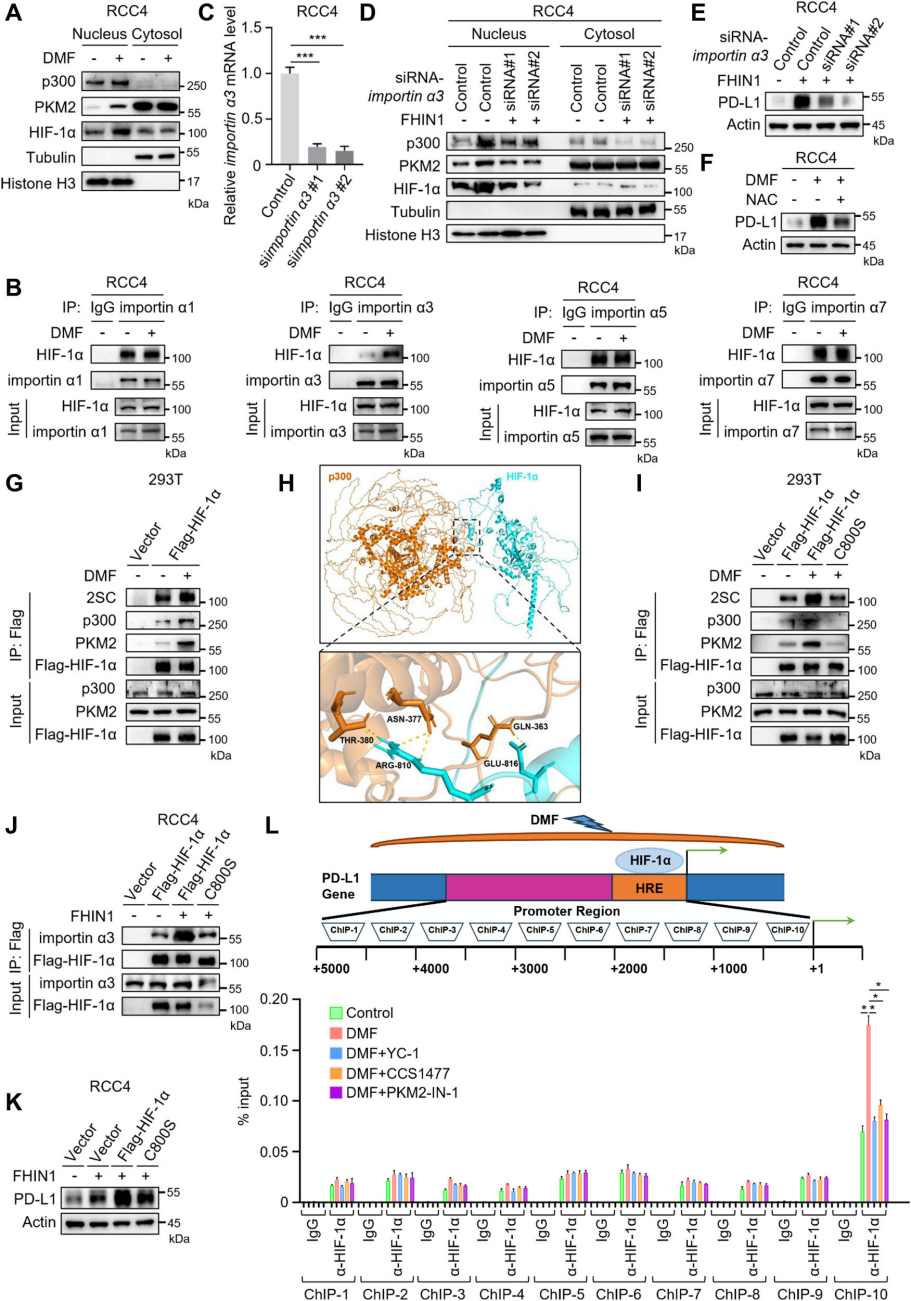
Fumarate promotes PD-L1 expression through succination and activation of HIF-1α. **A** Western blot analysis was conducted to examine the cytosolic and nuclear localization of p300, PKM2, and HIF-1α in RCC4 cells treated with DMF (50 μM, 12 h). **B** RCC4 cells were transfected with importin α1, importin α3, importin α5, and importin α7, both in the presence and absence of DMF. The binding interactions between Flag-HIF-1α and the four nuclear transport proteins were analyzed by co-immunoprecipitation (co-IP) and Western blot. **C** qRT-PCR analysis of *importin α3* transcription levels in RCC4 cells transfected with control or importin α3-targeting siRNAs. **D** After transfection with two siRNA fragments specifically designed to knock down importin α3 expression, RCC4 cells were treated with FHIN1 (20 μM, 24 h), followed by western blot analysis to determine the cytosolic and nuclear localization of p300, PKM2, and HIF-1α. **E** Following transfection of RCC4 cells with two siRNA fragments designed to knock down importin α3 expression, the cells were treated with FHIN1 (20 μM, 24 h), and PD-L1 expression levels were analyzed by western blot. **F** The effect of N-acetyl-L-cysteine (NAC) on DMF-induced PD-L1 degradation was determined by Western blot. RCC4 cells were pretreated with 5 mM NAC for 1 h before being treated with DMF (50 μM, 12 h). **G** The succination status of Flag-HIF-1α in HEK-293T cells transfected with Flag-HIF-1α, with or without DMF treatment, was evaluated by co-IP analysis, as well as its binding interactions with p300 and PKM2. **H** Computational molecular docking simulation predicts the interaction between HIF-1α and p300. **I** HEK-293T cells were transfected with Flag-HIF-1α WT or C800S mutant in the presence or absence of DMF. Succination levels of Flag-HIF-1α WT and C800S mutant were analyzed by co-IP and western blot, as well as their binding interactions with p300 and PKM2. **J** In the presence or absence of FHIN1, RCC4 cells were transfected with Flag-HIF-1α wild-type or C800S mutant. The binding interactions between Flag-HIF-1α wild-type and C800S mutant with importin α3 were analyzed by co-IP and western blot. **K** In the presence or absence of FHIN1, RCC4 cells were transfected with Flag-HIF-1α WT or C800S mutant, and PD-L1 expression levels were analyzed by western blot. **L** Chromatin immunoprecipitation (ChIP) analysis of the PD-L1 promoter in RCC4 cells using anti-HIF-1α monoclonal antibody (mAb) was conducted. The experiments were performed in triplicates and repeated three times.

To investigate how fumarate regulates the transcriptional activity of the HIF-1α/p300/PKM2 complex, we performed western blot analysis on nuclear and cytoplasmic fractions of endogenous HIF-1α, p300, and PKM2. The results showed that DMF treatment enhanced the nuclear translocation of these components to varying extents (Fig. 5A). Previous studies have reported that the nuclear translocation of HIF-1α is mediated by nuclear transport receptors importin α1, α3, α5, and α7 [30]. To determine which importin mediates the nuclear translocation of HIF-1α under DMF treatment, we conducted co-immunoprecipitation experiments. The results indicated that DMF treatment specifically increased the interaction between HIF-1α and importin α3 in RCC4 cells (Fig. 5B). Additionally, knockdown of importin α3 effectively blocked FHIN1-induced nuclear translocation of HIF-1α, p300, and PKM2 (Fig. 5C, D), as well as PD-L1 expression (Fig. 5E). These findings highlight the crucial role of importin α3 in fumarate-induced regulation of the HIF-1α/p300/PKM2 complex and subsequent PD-L1 expression.

As a thiol-reactive electrophile, DMF can modify nucleophilic cysteine residues on proteins to form S-(2-succinyl) cysteine (2SC) fumarate, a process known as succination [22]. To investigate whether succination is involved in fumarate-mediated PD-L1 expression, we pretreated cells with N-acetyl-L-cysteine (NAC), a cell-permeable thiol that competes with cysteine for succination, prior to DMF treatment. Notably, NAC pretreatment abolished the upregulatory effect of DMF on PD-L1 expression (Fig. 5F), suggesting that HIF-1α may undergo succination, with NAC preventing this modification. Using an antibody specific to 2SC, we confirmed that DMF treatment significantly increased the succination levels of HIF-1α (Fig. 5G). Moreover, DMF enhanced the binding of HIF-1α to p300 and PKM2 (Fig. 5G). We next identified the cysteine residue in HIF-1α that is succinated and regulates its interaction with p300 and PKM2. Previous studies have pinpointed residues essential for HIF-1α‘s interaction with p300, including cysteine 800 (C800) [31]. Intriguingly, among the four HIF-1α residues critical for p300 recruitment, one is the cysteine 800 (C800) site [31]. Docking analysis indicated that residues near C800 are crucial for HIF-1α‘s binding to p300 (Fig. 5H), suggesting that succination at C800 might influence this interaction. To test this hypothesis, we mutated C800 to serine (C800S), which prevents succination. This mutation significantly reduced HIF-1α succination under DMF treatment and decreased its binding to p300, PKM2, and importin α3 (Fig. 5I, J). Furthermore, the C800S mutation effectively blocked FHIN1-induced upregulation of PD-L1 expression (Fig. 5K). Given that HIF-1α directly binds to the promoters of target genes [32], we used ChIP-qPCR to verify whether PD-L1 is a direct target of HIF-1α upon DMF treatment. We found that HIF-1α interacts with the PD-L1 promoter (~0.5 kb proximal to the transcription start site) (Fig. 5L). Of note, DMF treatment significantly increased HIF-1α binding to PD-L1 promoter, while inhibitors targeting the HIF-1α/p300/PKM2 transcriptional complex decreased this binding (Fig. 5L). These results indicate that fumarate upregulates PD-L1 expression by succinating HIF-1α and enhancing the activity of the HIF-1α/p300/PKM2 complex.

### DMF enhances the efficacy of immunotherapy in renal cancer

**Fig. 6:**
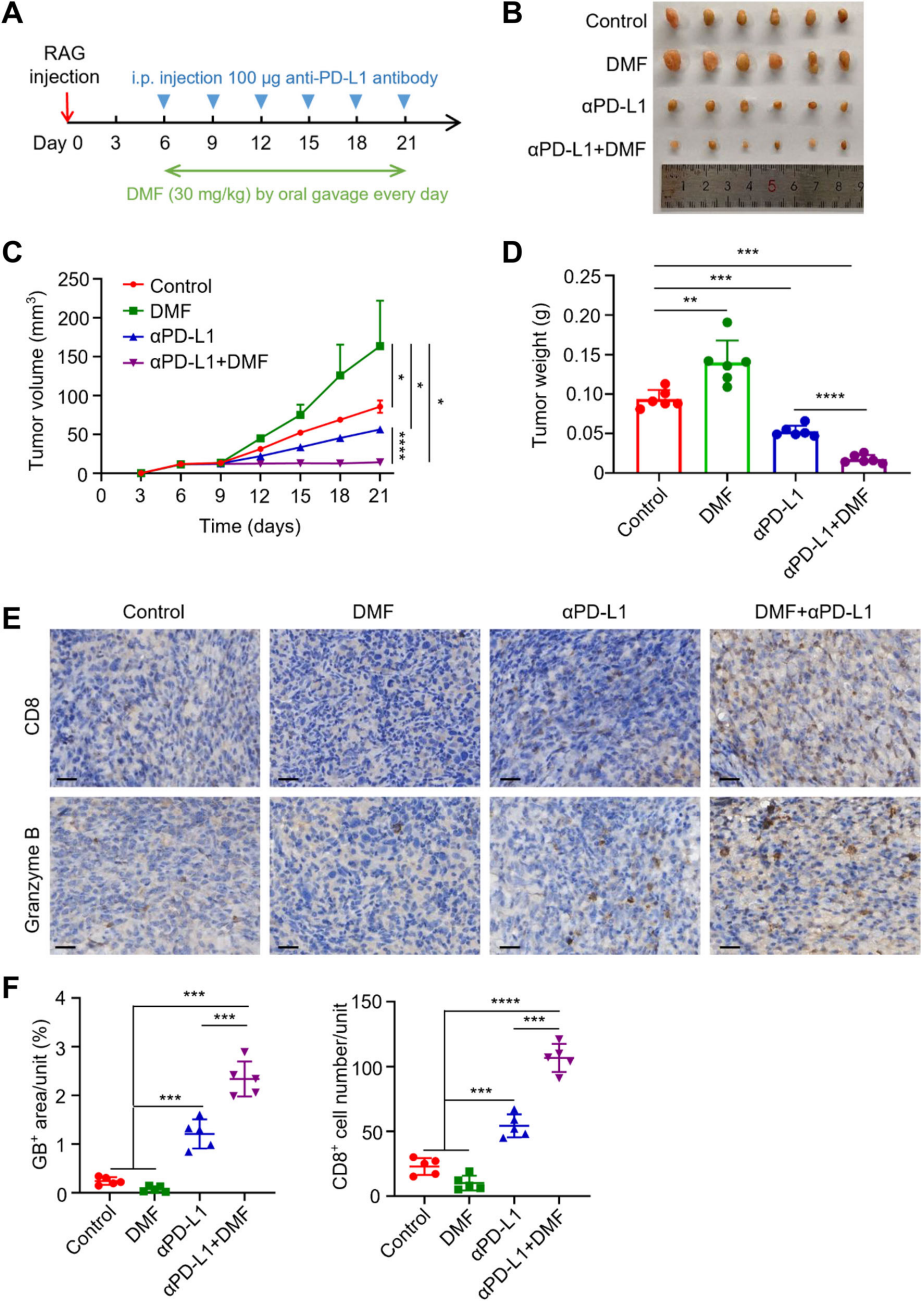
DMF synergizes with anti-PD-L1 antibodies to inhibit the tumor growth in renal cancer. **A** A schematic representation of the animal experiment process is shown. **B** Female BALB/c mice underwent treatment with a control, DMF, αPD-L1, or a combination of DMF + αPD-L1. Tumors were subsequently resected from each treatment group as indicated, with n = 6 mice per group. **C** Tumor growth of RAG cells in female BALB/c mice was determined. Statistical differences were determined by ordinary one-way ANOVA. *P < 0.05, ****P < 0.0001. **D** The weight of tumors resected from each group of mice that received different treatments as indicated was analyzed. Data represent mean ± SD, n = 6 mice per group. Statistical differences were determined by ordinary one-way ANOVA. **P < 0.01, ***P < 0.001, ****P < 0.0001. **E** Immunohistochemistry showed CD8^+^ T cell infiltration and granzyme B expression in the RAG tumor tissues as indicated (scale bars, 20 μm). **F** Quantifications of images in (**E**). Data represent mean ± SD from six independent samples of each group. Statistical differences were determined by ordinary one-way ANOVA. ***P < 0.001, ****P < 0.0001.

Inhibition of PD-L1 in tumor cells can activate anti-tumor immunity, but not all patients respond to this treatment [13]. Factors such as the tumor mutation burden (TMB), tumor microenvironment (TME), and the patient’s immune status can influence the therapeutic effect of PD-L1 antibodies [15]. One clinical strategy to increase the efficacy of immunotherapy is to increase the abundance of PD-L1 in tumor cells, and then combine it with anti-PD-L1 antibodies. Given that DMF is a widely used clinical drug, we investigated whether DMF could serve as an adjuvant to enhance the therapeutic efficacy of immunotherapy in renal cancer. We first confirmed that DMF can upregulate PD-L1 expression in RAG cells, a mouse renal cancer cell line, through the HIF-1α/p300/PKM2 transcriptional complex (Supplementary Fig. 3A–D). We then conducted an in vivo experiment where RAG cells were inoculated into the right posterior region of BALB/c mice, and the mice were treated with DMF and anti-PD-L1 antibodies (Fig. 6A). The results showed that tumor size increased after DMF administration, consistent with in vitro observations, suggesting that DMF can promote tumor immune evasion (Fig. 6B–D). Additionally, anti-PD-L1 antibody treatment reduced tumor growth and tumor weight (Fig. 6B–D). More importantly, the combination of DMF and anti-PD-L1 antibodies yielded the best therapeutic effect (Fig. 6B–D). Immunohistochemical (IHC) analysis of tumor tissues revealed that combined DMF and anti-PD-L1 treatment significantly increased the number of tumor-infiltrating CD8^+^ T cells and the level of granzyme B, demonstrating a synergistic effect in stimulating CD8^+^ T cell tumor infiltration and anti-tumor immunity (Fig. 6E, F). In summary, the combined treatment of DMF and anti-PD-L1 antibodies significantly inhibits tumor growth, indicating that DMF can enhance the efficacy of immune checkpoint blockade therapy in renal cancer.

## Discussion

**Fig. 7:**
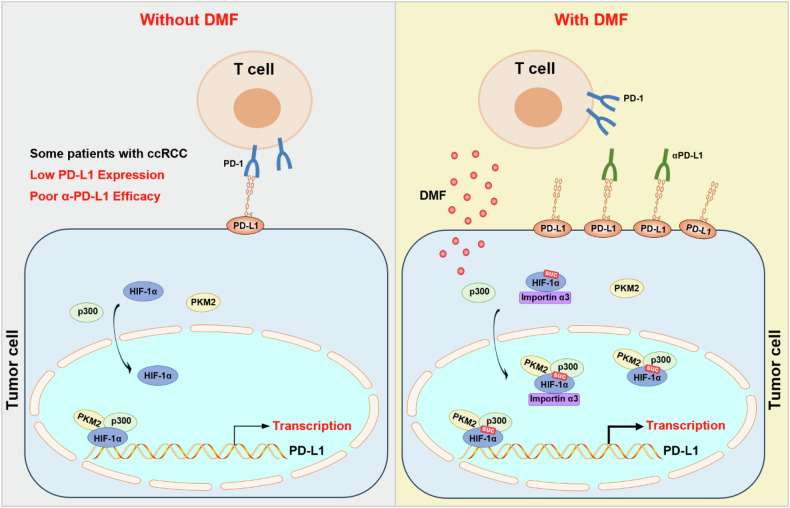
Fumarate upregulates PD-L1 expression through succination and activation of HIF-1α, thereby promoting immune evasion in ccRCC. In some patients with ccRCC, low levels of PD-L1 expression result in suboptimal clinical responses to anti-PD-L1 therapy (left panel). However, treatment with DMF upregulates PD-L1 expression, relying on the HIF-1α/p300/PKM2 transcriptional complex. Specifically, DMF induces succination of HIF-1α, which then directly interacts with the PD-L1 promoter region, regulated by the nuclear transport receptor importin α3. Importantly, combining DMF with anti-PD-L1 therapy enhances the efficacy of immunotherapy.

FH is an enzyme of the TCA cycle, and its deficiency or inactivation can lead to significant fumarate accumulation in the tumor stroma [33–36]. FH deficiency is associated with the development of certain types of kidney cancer, with mutations in the FH gene resulting in a highly invasive and metastatic phenotype with poor prognosis [37]. ccRCC, as one of the most common subtypes of RCC, also accounts for most RCC-associated deaths [10]. In this study, we found that FH is markedly downregulated in ccRCC, with its expression level negatively correlating with tumor stages and positively correlating with patient prognosis. In line with our results, a previous study found that over 70% of ccRCC samples showed decreased FH expression, with hypermethylation potentially playing a role in FH downregulation [38]. Additionally, a review noted that FH transcriptional downregulation has been observed in ccRCC and proposed that FH loss may be linked to hypermethylation and suppression of the tumor suppressor CDKN2A [39]. Together, hypermethylation maybe involved in the downregulation of FH in ccRCC. Mechanistically, we found that fumarate accumulation due to FH inhibition promotes succination of HIF-1α at C800, leading to its nuclear localization, recruitment of p300 and PKM2, and activation of PD-L1 expression (Fig. 7).

Immune surveillance evasion is a prominent hallmark of cancer [40]. The strategy of ICB for treating ccRCC has garnered considerable attention and the application of ICI has demonstrated the potential to improve the survival rates of tumor patients [41]. Additionally, various immunomodulatory molecules, such as PD1-IL2v, GM-CSF, and IL-2, have been implemented in clinical practice, further enhancing the efficacy of immunotherapy [42–44]. However, the development of new drugs often entails significant time and financial investments, and may encounter challenges such as low efficacy and side effects during clinical trials [45]. Consequently, the reutilization of existing drugs has sparked a wave of enthusiasm in recent years. For instance, aspirin, primarily known for its fever-reducing and analgesic properties, has shown to possess potential applications in immunotherapy for hepatocellular carcinoma (HCC) [46]. Similarly, metformin, a first-line treatment for type 2 diabetes mellitus, has also been discovered to possess anti-inflammatory and immunomodulatory properties [47]. DMF has been used to treat autoimmune diseases, and recently, DMF was also reported to regulate tumor metabolism and progression [25, 48]. In this study, we found that DMF can promote PD-L1 expression. Surprisingly, DMF only regulates the expression of PD-L1 in ccRCC. Considering that a key marker of ccRCC is the inactivating mutation of VHL, leading to the accumulation of HIF-1/2α and reprogramming of tumor metabolism [27], we examined the effect of DMF on HIF-1/2α. Our results indicate that DMF upregulates PD-L1 expression in ccRCC through the succination of HIF-1α rather than HIF-2α, a process dependent on the HIF-1α/p300/PKM2 transcriptional complex (Figs. 3 and 4). More importantly, we demonstrated that DMF can enhance the efficacy of immunotherapy, suggesting that even traditional drugs like DMF may have novel therapeutic uses (Fig. 7).

Clinical trials using anti-PD-L1 antibodies for ccRCC treatment have shown low response rates, likely due to the low expression levels of PD-L1 in some patients [13, 15]. Consequently, immunotherapy using ICB for ccRCC remains a significant challenge [16]. This study provided experimental evidence that DMF can act as an immune “sensitizer”. As shown in Fig. 6, the combined use of DMF and anti-PD-L1 antibody significantly increased the number of tumor-infiltrating CD8^+^ T cells and the levels of granzyme B, thereby enhancing the efficacy of immunotherapy in mice. These findings indicate that the combination of DMF and anti-PD-L1 antibody represents a promising strategy for ccRCC treatment, including for refractory patients.

HIF-1α is regulated by several post-translational modifications. For example, the phosphorylation of HIF-1α can affect its stability [49], while SUMOylation can alter its interaction with other proteins [50]. In this study, we demonstrated for the first time that the oncometabolite fumarate can succinate and activate HIF-1α, leading to the upregulation of PD-L1 and immune evasion in ccRCC. We also explored the role of DMF as an adjuvant drug for immunotherapy in ccRCC, providing an experimental basis for its potential use as an immune modulator. In summary, this study identified a target, drug, and strategy to enhance immunotherapy for ccRCC. The role of fumarate in TME of ccRCC warrants further investigation, which may lead to improved therapeutic windows for ccRCC.

## Materials and methods

### Cell culture

RCC4, RCC10, HCCLM3, MDA-MB-231, A375, RAG, and HEK 293 T cells were cultured in DMEM supplemented with 10% fetal bovine serum (FBS) and 1% penicillin/streptomycin (P/S). RPMI 1640 containing 10% FBS and 1% P/S was used to culture H1299, CT26, and 4T1 cells. For cell transfection, plasmids were introduced using EZ-trans reagents (Life iLab, China) according to the manufacturer’s protocols. The drugs used in this study included DMF (TargetMol, 624-49-7), Fumarate hydratase-IN-1 (MCE, HY-100004), YC-1 (MCE, HY-14927), PX478 (MCE, HY-10231), TC-S 7009 (MCE, HY-18371), A-485 (MCE, HY-107455), CCS1477 (MCE, HY-111784), Shikonin (MCE, HY-N0822), PKM2-2N-1 (MCE, HY-103617), Actinomycin D (MCE, HY-17559), and Cycloheximide (Selleck, S7418).

### siRNA transfection

siRNA transfection was performed using jetPRIMER (Polyplus, 101000046). According to the manufacturer’s instructions, cells were incubated with the transfection complex for 6 h before replacing it with fresh media. After 48 h, cells were harvested for total RNA or protein extraction. Small interfering RNA (siRNAs) specifically targeting FH, HIF-1α, and HIF-2α mRNA were synthesized by GenePharma (Shanghai, China).

The following effective sequences were used: si*FH*#1, 5′-UCUUGGGCAGGAAUUUAGUGGUUAU-3′ and si*FH*#2, 5′-GCACUGCUGUUGGUACAGGUUUAAA-3′; si*HIF-1α*#1, 5′-GGGAUUAACUCAGUUUGAATT-3′ and si*HIF-1*α#2, 5′-GCCGAGGAAGAACUAUGAATT-3′; si*HIF-2*α#1, 5′-ACGUAACGAUUUCAUGAAAT-3′ and si*HIF-2*α#2, 5′-CAACGUAACGAUUUCAUGAAA-3′; si*importin α3*#1, 5′-GGCGGAACAUUUGGUUUCATT-3′ and si*importin α3*#2, 5′-GCCACCAGGAAGUUAAAGUTT-3′.

### Western blot

Cells were washed with phosphate-buffered saline (PBS) and lysed in NP-40 lysis buffer (150 mM NaCl and 50 mM Tris-HCl, pH 7.5) containing 1% phosphatase inhibitor and 1% protease inhibitor at 4 °C for 40 min. The cell lysates were then heated with SDS-PAGE sample buffer at 100 °C for 15 min, followed by a standard Western blot procedure to detect specific proteins. Briefly, proteins were separated by electrophoresis on a 7.5–12.5% SDS-polyacrylamide gel and subsequently transferred to a nitrocellulose membrane. The membrane was incubated with the primary antibody overnight at 4 °C. After three washes with TBST, the secondary antibody conjugated with horseradish peroxidase (HRP) was added and incubated at room temperature for 1 h. Subsequently, the membrane was washed again with TBST. The protein bands were visualized using the Chemiluminescent HRP Substrate (Millipore) on the Chemiluminescent Imaging System (Tanon Science and Technology Co., Ltd) and analyzed by chemiluminescence and densitometry. All antibodies used for Western blot are commercially available and include Actin (Proteintech, 81115-1-RR), HIF-1α (Huabio, ER1802-41), HIF-1α (Cell Signaling Technology, 36169S), HIF-2α (Cell Signaling Technology, 59973S), p300 (Cell Signaling Technology, 54062S), PKM2 (Cell Signaling Technology, 4053S), Tubulin (Proteintech, 66031-1-Ig), Flag (GNI, GNI4110-FG), Histone H3 (Huabio, M1309-1), PD-L1 (Thermo, PA5-86027), PD-L1 (Proteintech, 66248-1-Ig), 2SC (Discovery Antibodies, crb2005017), CD8 (Huabio, 0108-7), Granzyme B (Huabio, HA500252), importin α1(Proteintech, 10819-1-AP), importin α3 (Proteintech, 12463-1-AP), importin α5 (Proteintech, 18137-1-AP), importin α7 (Proteintech, 12366-2-AP), Mouse secondary antibody (SAB, L3032), and Rabbit secondary antibody (SAB, L3012).

### Quantitative real-time PCR

Total RNA was extracted from cells using the EZ-press RNA purification kit from EZ Bioscience. According to the manufacturer’s instructions, the purified RNA was converted into cDNA using the gDNA Remover and Reverse Transcription Master Mix (EZ Bioscience). Subsequently, qRT-PCR was performed on the Applied Biosystems 7300 Plus Sequence Detection System (Applied Biosystems). The expression levels of target mRNA genes were normalized to the β-actin gene expression. Data analysis was conducted using the comparative cycling threshold (Ct) method.

The following primers were used: *β-actin*: forward, 5′-GGCATAGAGGTCTTTACGGATGTC-3′, reverse, 5′-TATTGGCAACGAGCGGTTCC-3′; *FH*: forward, 5′-TGCAATAATGAAGGCAGCAG-3′, reverse, 5′-TGATCCAGTCTGCCATACCA-3′; *HIF-1*α: forward, 5′-GAACGTCGAAAAGAAAAGTCTCG-3′, reverse, 5′-CCTTATCAAGATGCGAACTCACA-3′; *HIF-2*α: forward, 5′-CGACCATGAGGAGATTCGTGAG-3′, reverse, 5′-CGTGCAGTGCAAGACCTTCCAG-3′; *PD-L1*: forward, 5′- TGGCATTTGCTGAACGCATTT-3′, reverse, 5′-TGCAGCCAGGTCTAATTGTTTT-3′; *importin α3*: forward, 5′-AAGCAGTAGTTCAGTCCAAT-3′, reverse, 5′-GCCAGCATCAGTAAGGTAA-3′.

### ChIP assay

Chromatin immunoprecipitation (ChIP) assays were performed using lysates from MSC-1 cells, utilizing the Simple ChIP enzymatic chromatin IP kit (Cell Signaling Technology).

SYBR Green RT-qPCR was conducted using primers from previously reported references, specifically: hPDL1-chip-1, forward, 5′-TGTACTTACCTTCGAGTCTCT-3′, reverse, 5′-CTGAGGCTTGCTATTAACCA-3′; hPDL1-chip-2, forward, 5′-GTGTGAGTATGTATCTTCCTTG-3′, reverse, 5′-AGTTGCCTGATGAATGTTCT- 3′; hPDL1-chip-3, forward, 5′-AGTCCTCAAGGCTCTTCA-3′, reverse, 5′-TTAGTTATGGTGGTCAGGAA-3′; hPDL1-chip-4, forward, 5′-TTGTTGGAGGTCTCATCTT-3′, reverse, 5′-CTCATCTGACACTCACAATC-3′; hPDL1-chip-5, forward, 5′-GGAAACAGAGGAAGAGAAATG-3′, reverse, 5′-AGTGGACCTGAAGAGATGT-3′; hPDL1-chip-6, forward, 5′-GAAGGAAGGATGGTACTGATA-3′, reverse, 5′-GGTCTTGGAGGTCAACATT-3′; hPDL1-chip-7, forward, 5′-ACACGAATCCTCACATTACT-3′, reverse, 5′-AATCATATCCTCCTAGATGGC-3′; hPDL1-chip-8, forward, 5′-TTCGGGAACTTTGGGAAG-3′, reverse, 5′-GCTGACACTGCCTTGATT-3′; hPDL1-chip-9, forward, 5′-ATTATGACACCATCGTCTGT-3′, reverse, 5′-TCGTGGATTCTGTGACTTC-3′; hPDL1-chip-10, forward, 5′-CAGATGTTGGCTTGTTGTAA-3′, reverse, 5′-GTATCTAGTGTTGGTGTCCTA-3′.

### Immunohistochemistry (IHC)

The tumor sections underwent a series of processing steps. First, they were dewaxed and hydrated, followed by blocking of endogenous peroxidase activity using 3% hydrogen peroxide. Subsequently, the sections were blocked with PBS containing goat serum for 30 min, and incubated overnight at 4 °C with the primary antibodies. After thorough washing with PBS three times, the sections were incubated with secondary antibodies and streptavidin-enzyme conjugate. The resulting complex was visualized using DAB. Finally, the slides were counterstained with hematoxylin to complete the process.

### Separation of nuclear and cytoplasmic proteins

The separation of nuclear and cytoplasmic proteins was meticulously performed using the nuclear and cytoplasmic protein extraction kit (P0027), strictly following the manufacturer’s instructions. During the utilization of reagent A and reagent B, it was crucial to add 1% phosphatase inhibitor and 1% protease inhibitor to preserve protein integrity throughout the extraction process. After successful separation, the proteins were analyzed by Western blot to comprehensively assess their composition and distribution within the nuclear and cytoplasmic compartments.

### T cell–mediated tumor cell killing assay

Peripheral blood mononuclear cells (PBMC) were isolated from healthy donors. To activate T cells, the PBMCs were stimulated with ImmunoCult Human CD3/CD28/CD2 T cell activator (25 μL/mL, Stemcell) for the initial 3 days. Following stimulation, the cells were cultured in ImmunoCult-XF T cell expansion medium (Stemcell) with IL-2 (10 ng/mL, PeproTech) for 7 days, according to the manufacturer’s guidelines. Prior to the co-culture experiment, the cells were divided into control and treatment groups. The RCC10 or RCC4 cells were pretreated with DMF, YC-1, CCS1477, or PKM2-2N-1 drugs and allowed to adhere to the plates overnight. Subsequently, the activated T cells were co-cultured with the tumor cells at a 10:1 ratio in DMEM/F12 medium, supplemented with 10% FBS, 1% P/S, anti-CD3 antibody (100 ng/mL, eBioscience, Thermo Scientific), and IL-2 (10 ng/mL). This co-culture was maintained for 48 h. After the co-culture period, cell debris was removed by washing with PBS. Viable cancer cells were then stained with crystal violet, washed with 33% (vol/vol) acetic acid, and quantified using a spectrophotometer at an optical density of 570 nm.

### Detection of cell surface PD-L1

The protocol for detecting cell surface PD-L1 was carried out as previously described. After pretreating the cells with DMF, YC-1, CCS1477, and PKM2-2N-1 drugs, they were washed with PBS and transferred to separate centrifuge tubes. The cells were then incubated on ice for 30 min in the dark with an APC-conjugated anti-human CD274 antibody (1:100 dilution, BioLegend). Following incubation, the cells were washed three times with 2 mL of Cell Staining Buffer (BioLegend). The stained cells were resuspended in 200 μL of staining buffer and analyzed using a BD FACSCelesta flow cytometer (BD Biosciences). The acquired data were processed with FlowJo software.

### DNA constructs and mutagenesis

To generate diverse HIF-1α mutants, site-directed mutagenesis PCR was performed using KOD Fx (TOYOBO) in strict accordance with the manufacturer’s instructions. Mutation-specific primers were used to amplify HIF-1α plasmids over 30 cycles. Following amplification, the products were treated with the DpnI enzyme (Takara) to remove any residual template DNA. The resulting plasmids were then transformed into NcmDH5-α competent cells (NCM Biotech) and plated on culture media containing ampicillin. The presence of the intended mutations was confirmed by sequencing multiple clones.

### Animal experiments

The animal experiments in this study were conducted in accordance with the ethical standards approved by the Department of Experimental Animal Science at Fudan University. Female BALB/c mice, aged 4–5 weeks, were procured from Hangzhou Qizhen Laboratory Animal. The mice were housed under strict specific-pathogen-free (SPF) conditions, provided with unrestricted access to food and water, and maintained on a 12-h light/dark cycle with the temperature controlled at 22–23 °C. Each mouse received an injection of 1 × 10^7^ RAG cells into the right flank. For PD-L1 antibody treatment, 100 μg of PD-L1 antibody was administered intraperitoneally every 3 days. DMF was administered by gavage at a daily dose of 30 mg/kg per mouse. Tumor growth was monitored every 3 days by measuring the length and width of the tumor with vernier calipers, and tumor volume was calculated using the formula: (length/2) × width^2^.

### Statistics

Statistical analysis was performed using GraphPad Prism 8 software. Data presented in bar graphs are shown as fold changes or percentages relative to the control, with standard deviations (SD) derived from three independent experiments. Student’s *t*-test was applied to normally distributed data, while one-way ANOVA with Tukey’s multiple comparisons was employed for the analysis of more than two groups. The log-rank test was utilized for analyzing mouse survival data. Statistical significance was determined with a threshold of *P* < 0.05, and results were indicated as follows: ns, nonsignificant, *P < 0.05, **P < 0.01, ***P < 0.001, and ****P < 0.0001.

## Supplementary information

Supplementary Information

aj-checklist

Original Data Files

## Data Availability

Further information and requests for reagents may be directed to and will be fulfilled by the author Lei Lv (lvlei@fudan.edu.cn).

## References

[1] Frew IJ, Thoma CR, Georgiev S, Minola A, Hitz M, Montani M, et al. pVHL and PTEN tumour suppressor proteins cooperatively suppress kidney cyst formation. EMBO J. 2008;27:1747–57. 18497742 10.1038/emboj.2008.96 PMC2435131

[2] Li Y, Lih TM, Dhanasekaran SM, Mannan R, Chen L, Cieslik M, et al. Histopathologic and proteogenomic heterogeneity reveals features of clear cell renal cell carcinoma aggressiveness. Cancer Cell. 2023;41:139–63.e17. 36563681 10.1016/j.ccell.2022.12.001 PMC9839644

[3] Larkin J, Goh XY, Vetter M, Pickering L, Swanton C. Epigenetic regulation in RCC: opportunities for therapeutic intervention? Nat Rev Urol. 2012;9:147–55. 22249190 10.1038/nrurol.2011.236

[4] Zhang X, Li S, He J, Jin Y, Zhang R, Dong W, et al. TET2 suppresses VHL deficiency-driven clear cell renal cell carcinoma by inhibiting HIF signaling. Cancer Res. 2022;82:2097–109. 35176127 10.1158/0008-5472.CAN-21-3013

[5] Tumkur Sitaram R, Landstrom M, Roos G, Ljungberg B. Significance of PI3K signalling pathway in clear cell renal cell carcinoma in relation to VHL and HIF status. J Clin Pathol. 2021;74:216–22. 32467322 10.1136/jclinpath-2020-206693

[6] Yang J, Wang K, Yang Z. Treatment strategies for clear cell renal cell carcinoma: past, present and future. Front Oncol. 2023;13:1133832. 37025584 10.3389/fonc.2023.1133832 PMC10070676

[7] Xu JX, Maher VE, Zhang L, Tang S, Sridhara R, Ibrahim A, et al. FDA approval summary: nivolumab in advanced renal cell carcinoma after anti-angiogenic therapy and exploratory predictive biomarker analysis. Oncologist. 2017;22:311–7. 28232599 10.1634/theoncologist.2016-0476 PMC5344649

[8] Lin E, Liu X, Liu Y, Zhang Z, Xie L, Tian K, et al. Roles of the dynamic tumor immune microenvironment in the individualized treatment of advanced clear cell renal cell carcinoma. Front Immunol. 2021;12:653358. 33746989 10.3389/fimmu.2021.653358 PMC7970116

[9] Makhov P, Joshi S, Ghatalia P, Kutikov A, Uzzo RG, Kolenko VM. Resistance to systemic therapies in clear cell renal cell carcinoma: mechanisms and management strategies. Mol Cancer Ther. 2018;17:1355–64. 29967214 10.1158/1535-7163.MCT-17-1299 PMC6034114

[10] Hsieh JJ, Purdue MP, Signoretti S, Swanton C, Albiges L, Schmidinger M, et al. Renal cell carcinoma. Nat Rev Dis Prim. 2017;3:17009. 28276433 10.1038/nrdp.2017.9 PMC5936048

[11] Zhang R, Yang Y, Dong W, Lin M, He J, Zhang X, et al. D-mannose facilitates immunotherapy and radiotherapy of triple-negative breast cancer via degradation of PD-L1. Proc Natl Acad Sci USA. 2022;119:e2114851119. 10.1073/pnas.2114851119 PMC8872783 35181605

[12] Chai F, Li P, Liu X, Zhou Z, Ren H. Targeting the PD-L1 cytoplasmic domain and its regulatory pathways to enhance cancer immunotherapy. J Mol Cell Biol. 2024;15:mjad070. 10.1093/jmcb/mjad070 PMC11193063 37993416

[13] Niu M, Liu Y, Yi M, Jiao D, Wu K. Biological characteristics and clinical significance of soluble PD-1/PD-L1 and exosomal PD-L1 in cancer. Front Immunol. 2022;13:827921. 35386715 10.3389/fimmu.2022.827921 PMC8977417

[14] Wang H, Yao H, Li C, Shi H, Lan J, Li Z, et al. HIP1R targets PD-L1 to lysosomal degradation to alter T cell-mediated cytotoxicity. Nat Chem Biol. 2019;15:42–50. 30397328 10.1038/s41589-018-0161-x

[15] Doroshow DB, Bhalla S, Beasley MB, Sholl LM, Kerr KM, Gnjatic S, et al. PD-L1 as a biomarker of response to immune-checkpoint inhibitors. Nat Rev Clin Oncol. 2021;18:345–62. 33580222 10.1038/s41571-021-00473-5

[16] Deleuze A, Saout J, Dugay F, Peyronnet B, Mathieu R, Verhoest G, et al. Immunotherapy in renal cell carcinoma: the future is now. Int J Mol Sci. 2020;21:2532. 10.3390/ijms21072532 PMC7177761 32260578

[17] Walter B, Gil S, Naizhen X, Kruhlak MJ, Linehan WM, Srinivasan R, et al. Determination of the expression of PD-L1 in the morphologic spectrum of renal cell carcinoma. J Cancer. 2020;11:3596–603. 32284756 10.7150/jca.35738 PMC7150459

[18] Xiao WJ, Xu FJ, Zhang X, Zhou SX, Zhang HL, Dai B, et al. The prognostic value of programmed death-ligand 1 in a Chinese cohort with clear cell renal cell carcinoma. Front Oncol. 2019;9:879. 31824835 10.3389/fonc.2019.00879 PMC6886562

[19] Atkins MB, Tannir NM. Current and emerging therapies for first-line treatment of metastatic clear cell renal cell carcinoma. Cancer Treat Rev. 2018;70:127–37. 30173085 10.1016/j.ctrv.2018.07.009

[20] Cohan S, Kumar J, Arndorfer S, Zhu X, Zivkovic M, Tencer T. Comparative efficacy and safety of ozanimod and dimethyl fumarate for relapsing-remitting multiple sclerosis using matching-adjusted indirect comparison. CNS Drugs. 2021;35:795–804. 33847901 10.1007/s40263-021-00805-0 PMC8310468

[21] Bruck J, Dringen R, Amasuno A, Pau-Charles I, Ghoreschi K. A review of the mechanisms of action of dimethylfumarate in the treatment of psoriasis. Exp Dermatol. 2018;27:611–24. 29603404 10.1111/exd.13548

[22] Kornberg MD, Bhargava P, Kim PM, Putluri V, Snowman AM, Putluri N, et al. Dimethyl fumarate targets GAPDH and aerobic glycolysis to modulate immunity. Science. 2018;360:449–53. 29599194 10.1126/science.aan4665 PMC5924419

[23] Itoh K, Tong KI, Yamamoto M. Molecular mechanism activating Nrf2-Keap1 pathway in regulation of adaptive response to electrophiles. Free Radic Biol Med. 2004;36:1208–13. 15110385 10.1016/j.freeradbiomed.2004.02.075

[24] Vandermeeren M, Janssens S, Wouters H, Borghmans I, Borgers M, Beyaert R, et al. Dimethylfumarate is an inhibitor of cytokine-induced nuclear translocation of NF-kappa B1, but not RelA in normal human dermal fibroblast cells. J Investig Dermatol. 2001;116:124–30. 11168807 10.1046/j.1523-1747.2001.00211.x

[25] Schmitt A, Xu W, Bucher P, Grimm M, Konantz M, Horn H, et al. Dimethyl fumarate induces ferroptosis and impairs NF-kappaB/STAT3 signaling in DLBCL. Blood. 2021;138:871–84. 33876201 10.1182/blood.2020009404

[26] Yao X, Tan J, Lim KJ, Koh J, Ooi WF, Li Z, et al. VHL deficiency drives enhancer activation of oncogenes in clear cell renal cell carcinoma. Cancer Discov. 2017;7:1284–305. 28893800 10.1158/2159-8290.CD-17-0375

[27] Jonasch E, Walker CL, Rathmell WK. Clear cell renal cell carcinoma ontogeny and mechanisms of lethality. Nat Rev Nephrol. 2021;17:245–61. 33144689 10.1038/s41581-020-00359-2 PMC8172121

[28] Thompson CB. Into thin air: how we sense and respond to hypoxia. Cell. 2016;167:9–11. 27634319 10.1016/j.cell.2016.08.036

[29] Luo W, Hu H, Chang R, Zhong J, Knabel M, O’Meally R, et al. Pyruvate kinase M2 is a PHD3-stimulated coactivator for hypoxia-inducible factor 1. Cell. 2011;145:732–44. 21620138 10.1016/j.cell.2011.03.054 PMC3130564

[30] Depping R, Steinhoff A, Schindler SG, Friedrich B, Fagerlund R, Metzen E, et al. Nuclear translocation of hypoxia-inducible factors (HIFs): involvement of the classical importin alpha/beta pathway. Biochim Biophys Acta. 2008;1783:394–404. 18187047 10.1016/j.bbamcr.2007.12.006

[31] Freedman SJ, Sun ZY, Poy F, Kung AL, Livingston DM, Wagner G, et al. Structural basis for recruitment of CBP/p300 by hypoxia-inducible factor-1 alpha. Proc Natl Acad Sci USA. 2002;99:5367–72. 11959990 10.1073/pnas.082117899 PMC122775

[32] Ding XC, Wang LL, Zhang XD, Xu JL, Li PF, Liang H, et al. The relationship between expression of PD-L1 and HIF-1alpha in glioma cells under hypoxia. J Hematol Oncol. 2021;14:92. 34118979 10.1186/s13045-021-01102-5 PMC8199387

[33] Cheng J, Yan J, Liu Y, Shi J, Wang H, Zhou H, et al. Cancer-cell-derived fumarate suppresses the anti-tumor capacity of CD8(+) T cells in the tumor microenvironment. Cell Metab. 2023;35:961–78.e10. 37178684 10.1016/j.cmet.2023.04.017

[34] Marletta S, Marcolini L, Calio A, Pedron S, Antonini P, Martelli FM, et al. Stimulator of interferon genes (STING) immunohistochemical expression in fumarate hydratase-deficient renal cell carcinoma: biological and potential predictive implications. Virchows Arch. 2025;3:1–12. 10.1007/s00428-025-04041-5 PMC12546389 39899047

[35] Hooftman A, Peace CG, Ryan DG, Day EA, Yang M, McGettrick AF, et al. Macrophage fumarate hydratase restrains mtRNA-mediated interferon production. Nature. 2023;615:490–8. 36890227 10.1038/s41586-019-0000-0 PMC10411300

[36] Zecchini V, Paupe V, Herranz-Montoya I, Janssen J, Wortel IMN, Morris JL, et al. Fumarate induces vesicular release of mtDNA to drive innate immunity. Nature. 2023;615:499–506. 36890229 10.1038/s41586-023-05770-w PMC10017517

[37] Cancer Genome Atlas Research N, Linehan WM, Spellman PT, Ricketts CJ, Creighton CJ, Fei SS, et al. Comprehensive molecular characterization of papillary renal-cell carcinoma. N Engl J Med. 2016;374:135–45. 26536169 10.1056/NEJMoa1505917 PMC4775252

[38] Sudarshan S, Shanmugasundaram K, Naylor SL, Lin S, Livi CB, O’Neill CF, et al. Reduced expression of fumarate hydratase in clear cell renal cancer mediates HIF-2alpha accumulation and promotes migration and invasion. PLoS ONE. 2011;6:e21037. 21695080 10.1371/journal.pone.0021037 PMC3114862

[39] Schmidt C, Sciacovelli M, Frezza C. Fumarate hydratase in cancer: a multifaceted tumour suppressor. Semin Cell Dev Biol. 2020;98:15–25. 31085323 10.1016/j.semcdb.2019.05.002 PMC6974395

[40] Chen DS, Mellman I. Elements of cancer immunity and the cancer-immune set point. Nature. 2017;541:321–30. 28102259 10.1038/nature21349

[41] Miao D, Margolis CA, Gao W, Voss MH, Li W, Martini DJ, et al. Genomic correlates of response to immune checkpoint therapies in clear cell renal cell carcinoma. Science. 2018;359:801–6. 29301960 10.1126/science.aan5951 PMC6035749

[42] Gao Y, Yu L, Miao H. Bispecific immune molecule PD1-IL2v: a new therapeutic strategy for pancreatic ductal adenocarcinoma. Signal Transduct Target Ther. 2023;8:382. 37798307 10.1038/s41392-023-01611-4 PMC10556073

[43] Bhattacharya P, Budnick I, Singh M, Thiruppathi M, Alharshawi K, Elshabrawy H, et al. Dual role of GM-CSF as a pro-inflammatory and a regulatory cytokine: implications for immune therapy. J Interferon Cytokine Res. 2015;35:585–99. 25803788 10.1089/jir.2014.0149 PMC4529096

[44] Sim GC, Radvanyi L. The IL-2 cytokine family in cancer immunotherapy. Cytokine Growth Factor Rev. 2014;25:377–90. 25200249 10.1016/j.cytogfr.2014.07.018

[45] Prasad S, Gupta SC, Aggarwal BB. Serendipity in cancer drug discovery: rational or coincidence? Trends Pharm Sci. 2016;37:435–50. 27083322 10.1016/j.tips.2016.03.004

[46] Lin M, He J, Zhang X, Sun X, Dong W, Zhang R, et al. Targeting fibrinogen-like protein 1 enhances immunotherapy in hepatocellular carcinoma. J Clin Investig. 2023;133:e164528. 10.1172/JCI164528 PMC10145938 37115693

[47] Foretz M, Guigas B, Viollet B. Metformin: update on mechanisms of action and repurposing potential. Nat Rev Endocrinol. 2023;19:460–76. 37130947 10.1038/s41574-023-00833-4 PMC10153049

[48] Gonnella R, Zarrella R, Santarelli R, Germano CA, Gilardini Montani MS, Cirone M. Mechanisms of sensitivity and resistance of primary effusion lymphoma to dimethyl fumarate (DMF). Int J Mol Sci. 2022;23:6773. 10.3390/ijms23126773 PMC9223506 35743211

[49] Han HJ, Saeidi S, Kim SJ, Piao JY, Lim S, Guillen-Quispe YN, et al. Alternative regulation of HIF-1alpha stability through phosphorylation on Ser451. Biochem Biophys Res Commun. 2021;545:150–6. 33550096 10.1016/j.bbrc.2021.01.047

[50] Zhang J, Ouyang F, Gao A, Zeng T, Li M, Li H, et al. ESM1 enhances fatty acid synthesis and vascular mimicry in ovarian cancer by utilizing the PKM2-dependent Warburg effect within the hypoxic tumor microenvironment. Mol Cancer. 2024;23:94. 38720298 10.1186/s12943-024-02009-8 PMC11077861

