# Supplementary material for: Oncometabolite fumarate facilitates PD-L1 expression and immune evasion in clear cell renal cell carcinoma
Source: Cell Death Dis. 2025 Jun 3;16(1):432. doi: 10.1038/s41419-025-07752-4 (PMC12134299; doi:10.1038/s41419-025-07752-4)
Supplement: Supplementary file 1 — Supplementary Information [file 41419_2025_7752_MOESM1_ESM.pdf]

Supplemental Information

Oncometabolite fumarate facilitates PD-L1 expression and immune evasion in clear cell renal cell carcinoma

Yi Gao[1], Shiyin Fan[1], Xue Sun[1], Jiaxi Li[1], Yue Dai[1], Hongchen Li[2], Haijie Ma[3]*, Yanping Xu[2]*, Lei Lv[1]*

Supplemental Figure 1

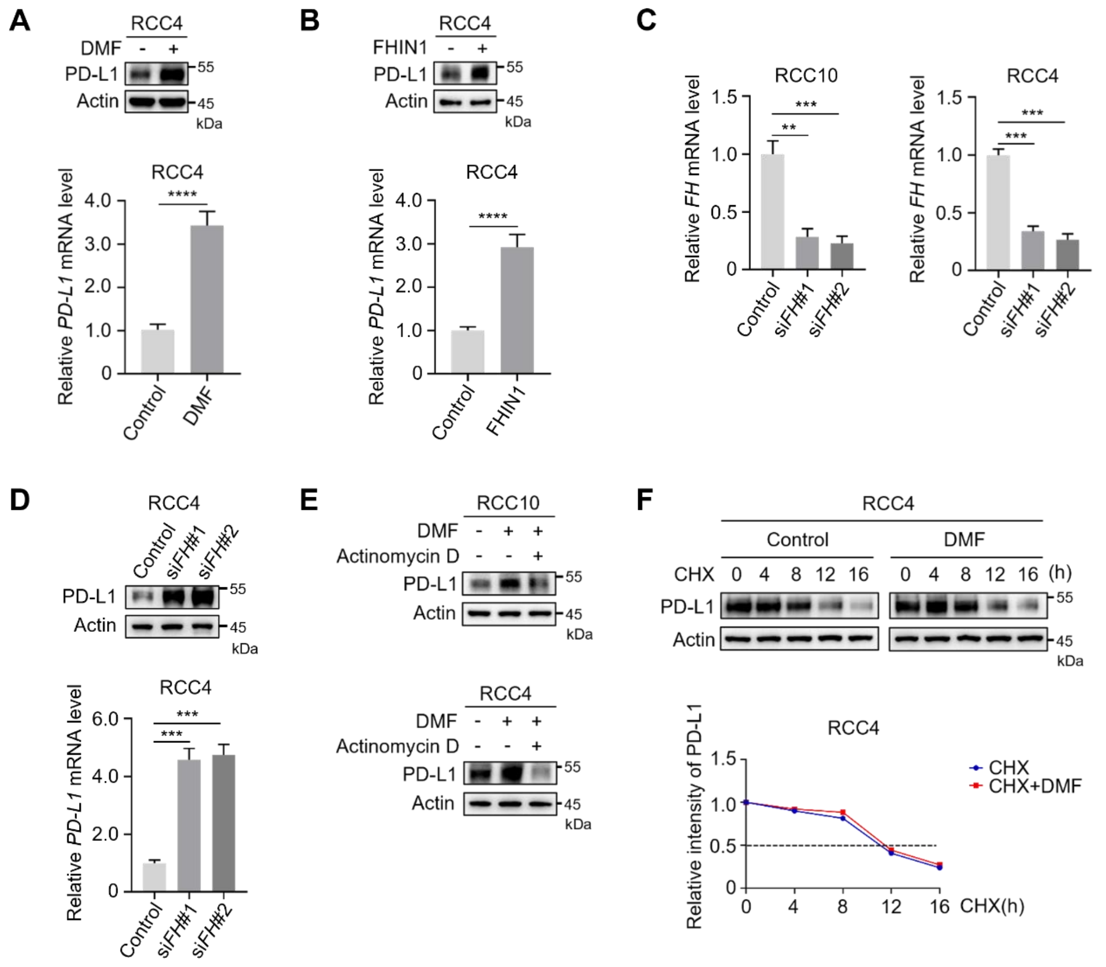

Supplemental Figure 1. Fumarate enhances PD-L1 expression through transcriptional regulation in ccRCC.

(**A**) Western blot and qRT-PCR analyses of PD-L1 expression in RCC4 cells, treated with or without DMF (50 μM, 12 h), were performed. ****P < 0.0001. **(B)** Western blot and qRT-PCR analyses of PD-L1 expression in RCC4 cells, treated with or without FHIN1 (20 μM, 24 h), were performed. ****P < 0.0001. (**C**) qRT-PCR analysis of FH transcription levels in RCC10 and RCC4 cells transfected with siRNAs targeting FH or

control. **$P < 0.01$, ***$P < 0.001$. **(D)** Western blot and qRT-PCR analysis of the PD-L1 expression in RCC4 cells transfected with siRNAs-targeting FH. ***$P < 0.001$. **(E)** Western blot analysis of PD-L1 levels in RCC10 and RCC4 cells treated with DMF (50 μM, 12 h) in the absence or presence of Actinomycin D (5 μg/mL, 24 h). **(F)** The half-life of PD-L1 under DMF (50 μM) treatment was determined by cycloheximide (CHX)-chase assay in RCC4 cells, with line charts shown below. Values are means ± SD from n = 3 independent experiments. Statistical differences were determined by Student's *t*-test.

Supplemental Figure 2

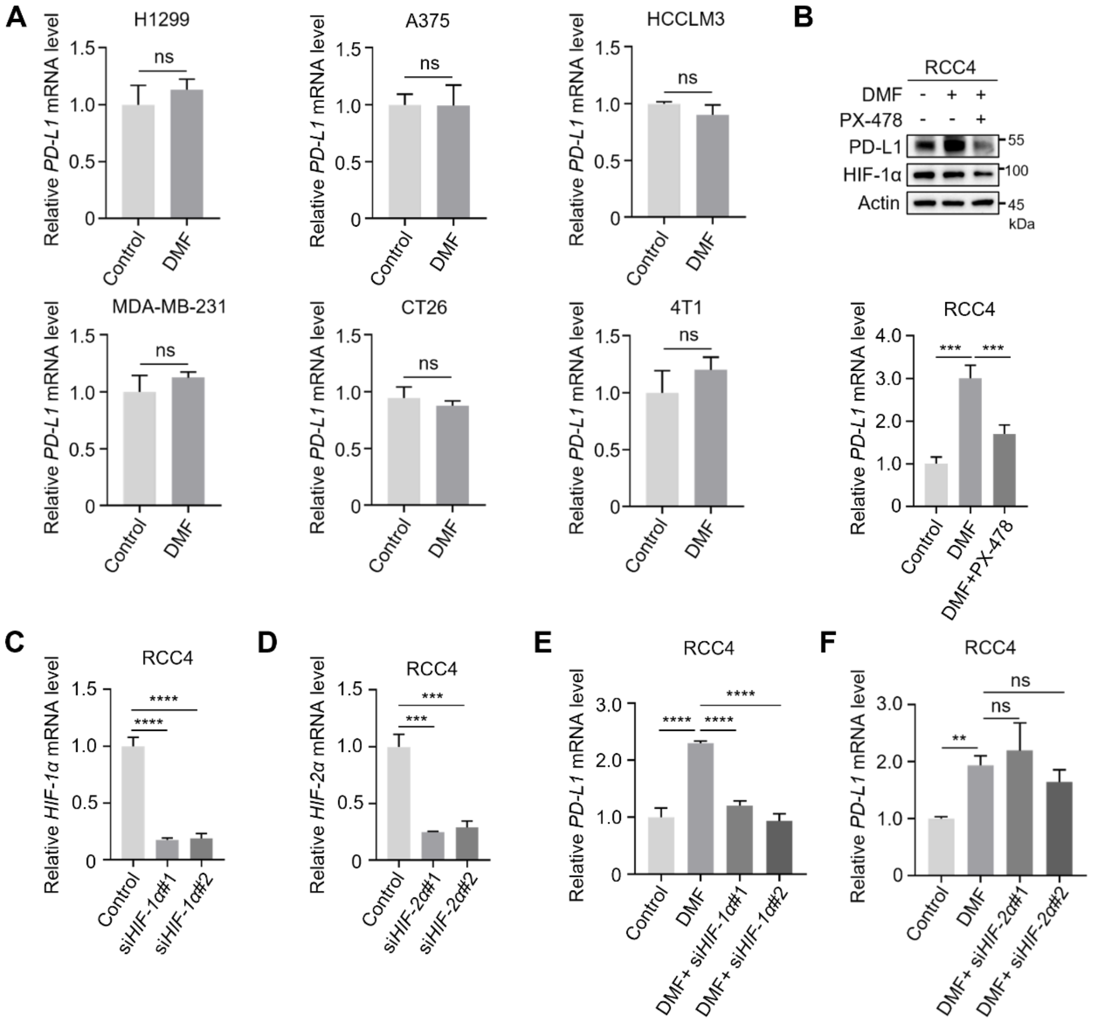

Supplemental Figure 2. Fumarate upregulates PD-L1 expression through regulation of HIF-1α/p300/PKM2 in ccRCC.

(A) qRT-PCR analyses were conducted to assess the mRNA expression levels in four human tumor cell lines (non-small cell lung cancer H1299, melanoma A375, liver cancer HCCLM3, breast cancer MDA-MB-231) and two mouse tumor cell lines (colorectal cancer CT26, breast cancer 4T1) after treatment with 50 μM DMF for 12

hours. ns, nonsignificant. **(B)** Western blot and qRT-PCR analyses were performed to assess the PD-L1 levels in RCC4 cells treated with DMF, both with and without 20 μM PX-478, a HIF-1α inhibitor. ***P < 0.001. **(C)** qRT-PCR analysis of *HIF-1α* transcription levels in RCC4 cells transfected with siRNAs-targeting *control*, *HIF-1α*. ****P < 0.0001. **(D)** qRT-PCR analysis of *HIF-2α* transcription levels in RCC4 cells transfected with siRNAs-targeting *control*, *HIF-2α*. ***P < 0.001. **(E)** Following transfection with two siRNA fragments designed to knockdown HIF-1α expression, RCC4 cells were treated with DMF, followed by the detection of PD-L1 expression levels through qRT-PCR techniques. ****P < 0.0001. **(F)** After transfection of RCC4 cells with two HIF-2α-knockdown siRNAs, treatment with DMF was followed by detection of PD-L1 expression using qRT-PCR techniques. ns, nonsignificant, **P < 0.01.

Supplemental Figure 3

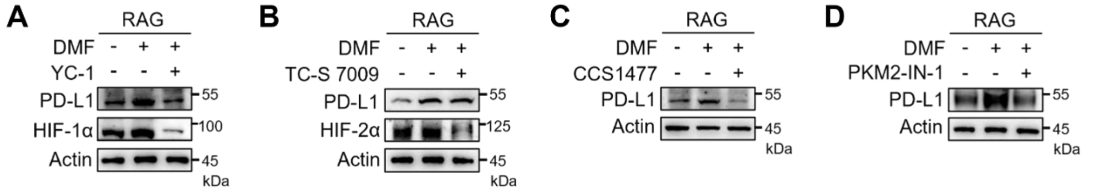

Supplemental Figure 3. DMF-induced PD-L1 expression depends on HIF-1α/ p300/PKM2, rather than HIF-2α in RAG cells.

(A) Western blot analysis was performed to assess the PD-L1 levels in RAG cell treated with DMF, both with and without 5 µM YC-1, a HIF-1α inhibitor. **(B)** Western blot analysis was performed to assess PD-L1 levels in RAG cells treated with DMF, with or without 50 µM TC-S 7009, an inhibitor of HIF-2α. **(C)** Western blot analysis was performed to assess the PD-L1 levels in RAG cells treated with DMF, both in the absence and presence of 100 nM CCS1477, a p300 inhibitor. **(D)** Western blot analysis was performed to assess PD-L1 levels in RAG cells that were treated with DMF, with or without the addition of 0.3 µM PKM2-IN-1, a PKM2 inhibitor.
