# Supplementary material for: Oncometabolite fumarate facilitates PD-L1 expression and immune evasion in clear cell renal cell carcinoma
Source: Cell Death Dis. 2025 Jun 3;16(1):432. doi: 10.1038/s41419-025-07752-4 (PMC12134299; doi:10.1038/s41419-025-07752-4)

Figure 2

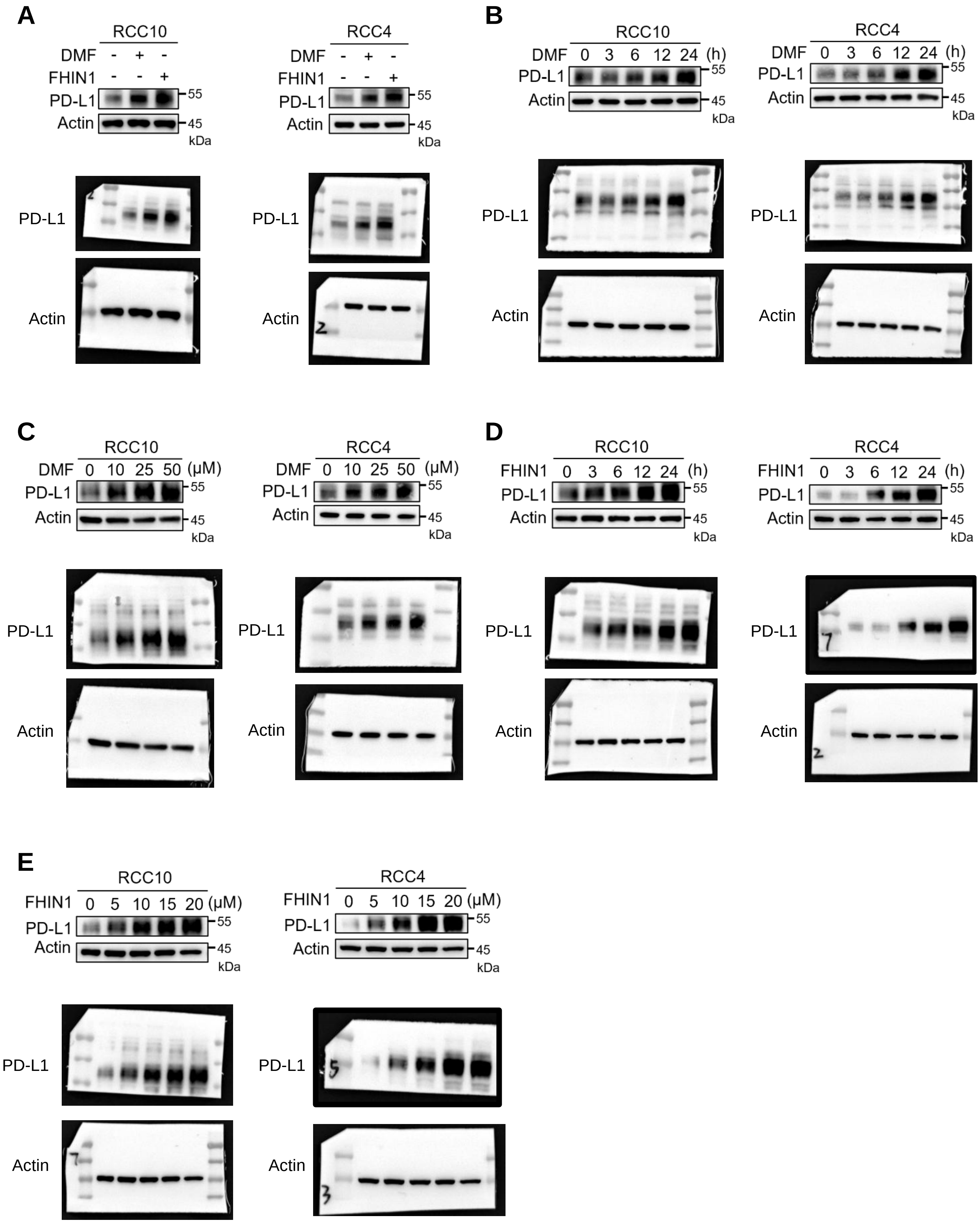
A
RCC10
DMF - + -
FHIN1 - - +
PD-L1
Actin
55
45
kDa
RCC4
DMF - + -
FHIN1 - - +
PD-L1
Actin
55
45
kDa
PD-L1
Actin
PD-L1
Actin
B
RCC10
DMF 0 3 6 12 24 (h)
PD-L1
Actin
55
45
kDa
RCC4
DMF 0 3 6 12 24 (h)
PD-L1
Actin
55
45
kDa
PD-L1
Actin
PD-L1
Actin
C
RCC10
DMF 0 10 25 50 (μM)
PD-L1
Actin
55
45
kDa
RCC4
DMF 0 10 25 50 (μM)
PD-L1
Actin
55
45
kDa
PD-L1
Actin
PD-L1
Actin
D
RCC10
FHIN1 0 3 6 12 24 (h)
PD-L1
Actin
55
45
kDa
RCC4
FHIN1 0 3 6 12 24 (h)
PD-L1
Actin
55
45
kDa
PD-L1
Actin
PD-L1
Actin
E
RCC10
FHIN1 0 5 10 15 20 (μM)
PD-L1
Actin
55
45
kDa
RCC4
FHIN1 0 5 10 15 20 (μM)
PD-L1
Actin
55
45
kDa
PD-L1
Actin
PD-L1
Actin

Figure 3

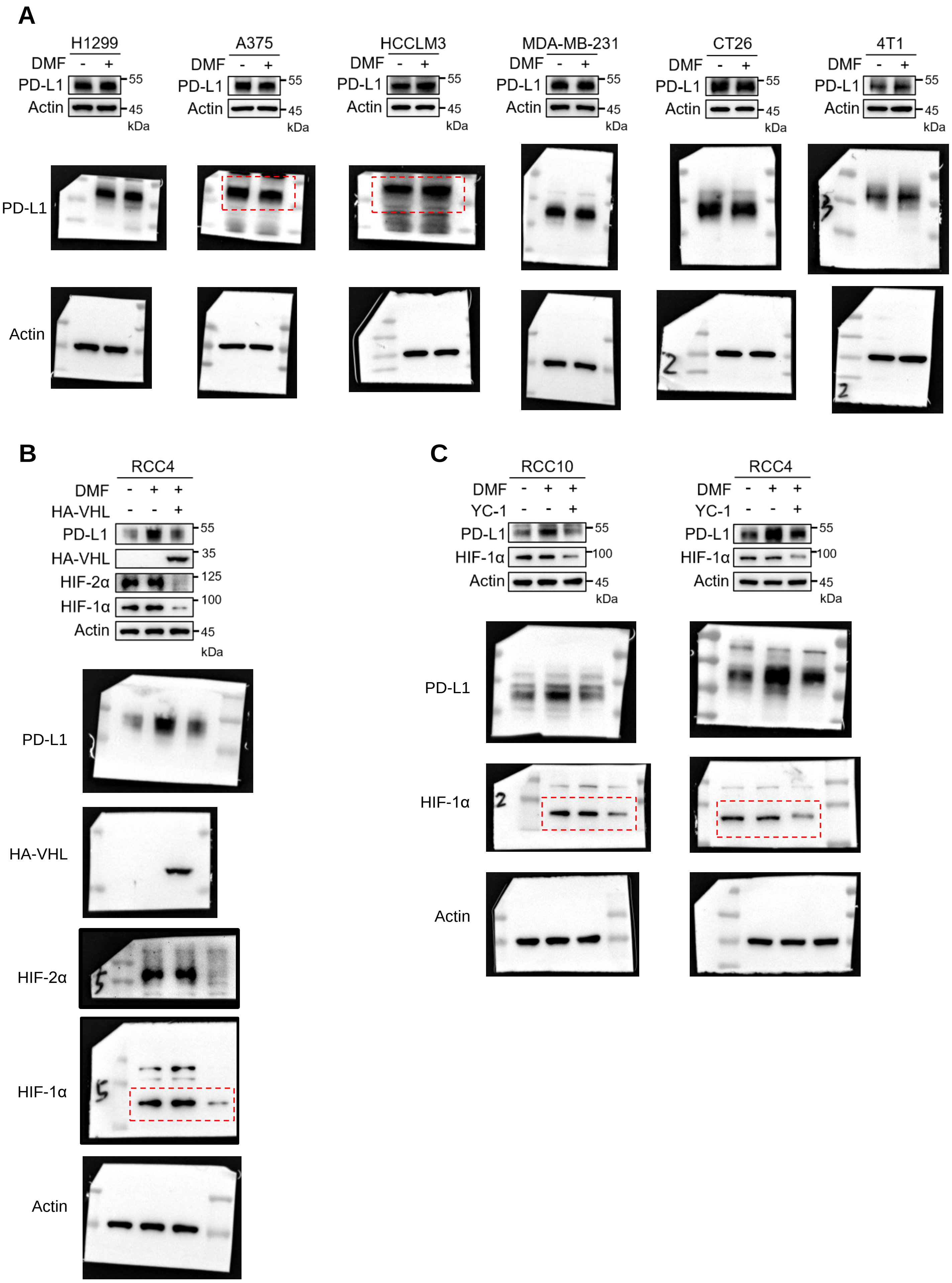
A
H1299
A375
HCCLM3
MDA-MB-231
CT26
4T1
DMF - +
PD-L1
Actin
55
45
kDa
B
RCC4
DMF - + +
HA-VHL - - +
PD-L1
HA-VHL
HIF-2α
HIF-1α
Actin
55
35
125
100
45
kDa
C
RCC10
RCC4
DMF - + +
YC-1 - - +
PD-L1
HIF-1α
Actin
55
100
45
kDa

Figure 3

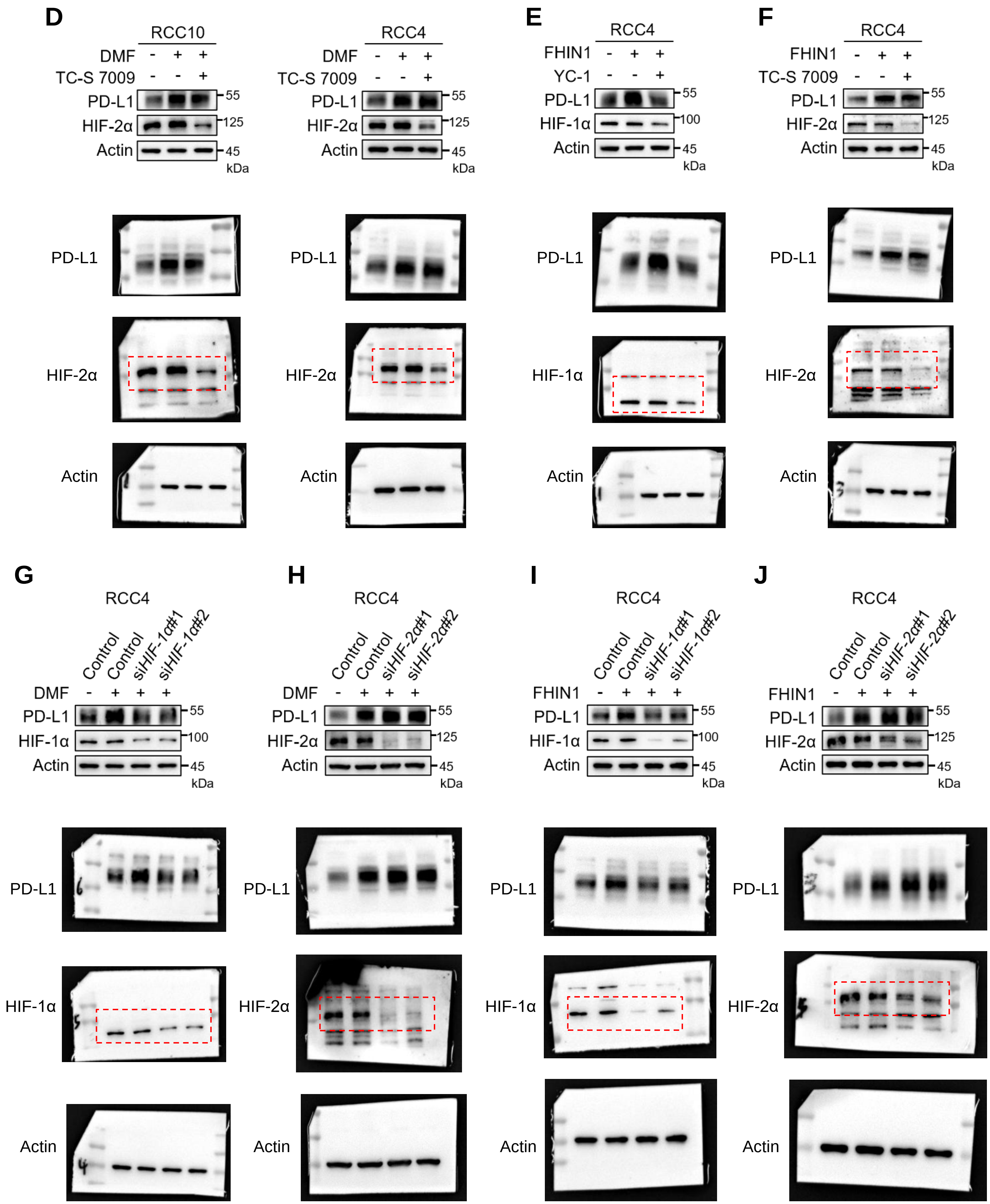

D
RCC10
DMF - + +
TC-S 7009 - - +
PD-L1
HIF-2α
Actin
55
125
45
kDa
RCC4
DMF - + +
TC-S 7009 - - +
PD-L1
HIF-2α
Actin
55
125
45
kDa
E
RCC4
FHIN1 - + +
YC-1 - - +
PD-L1
HIF-1α
Actin
55
100
45
kDa
F
RCC4
FHIN1 - + +
TC-S 7009 - - +
PD-L1
HIF-2α
Actin
55
125
45
kDa
G
RCC4
Control
Control
siHIF-1α#1
siHIF-1α#2
DMF - + + +
PD-L1
HIF-1α
Actin
55
100
45
kDa
H
RCC4
Control
Control
siHIF-2α#1
siHIF-2α#2
DMF - + + +
PD-L1
HIF-2α
Actin
55
125
45
kDa
I
RCC4
Control
Control
siHIF-1α#1
siHIF-1α#2
FHIN1 - + + +
PD-L1
HIF-1α
Actin
55
100
45
kDa
J
RCC4
Control
Control
siHIF-2α#1
siHIF-2α#2
FHIN1 - + + +
PD-L1
HIF-2α
Actin
55
125
45
kDa

Figure 4

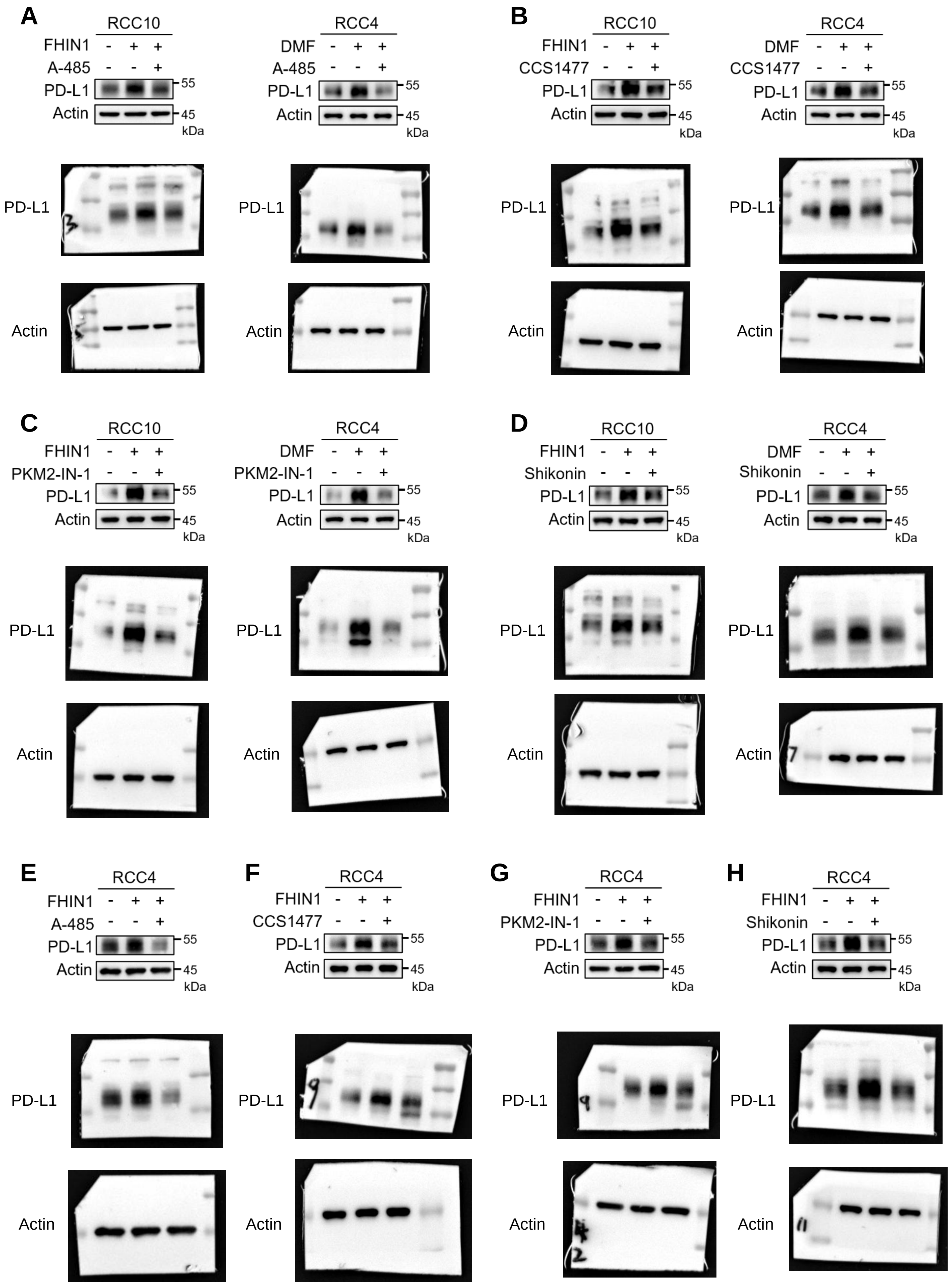
A
RCC10
FHIN1 - + +
A-485 - - +
PD-L1
Actin
55
45
kDa
RCC4
DMF - + +
A-485 - - +
PD-L1
Actin
B
RCC10
FHIN1 - + +
CCS1477 - - +
RCC4
DMF - + +
CCS1477 - - +
C
RCC10
FHIN1 - + +
PKM2-IN-1 - - +
RCC4
DMF - + +
PKM2-IN-1 - - +
D
RCC10
FHIN1 - + +
Shikonin - - +
RCC4
DMF - + +
Shikonin - - +
E
RCC4
FHIN1 - + +
A-485 - - +
F
RCC4
FHIN1 - + +
CCS1477 - - +
G
RCC4
FHIN1 - + +
PKM2-IN-1 - - +
H
RCC4
FHIN1 - + +
Shikonin - - +

Figure 5

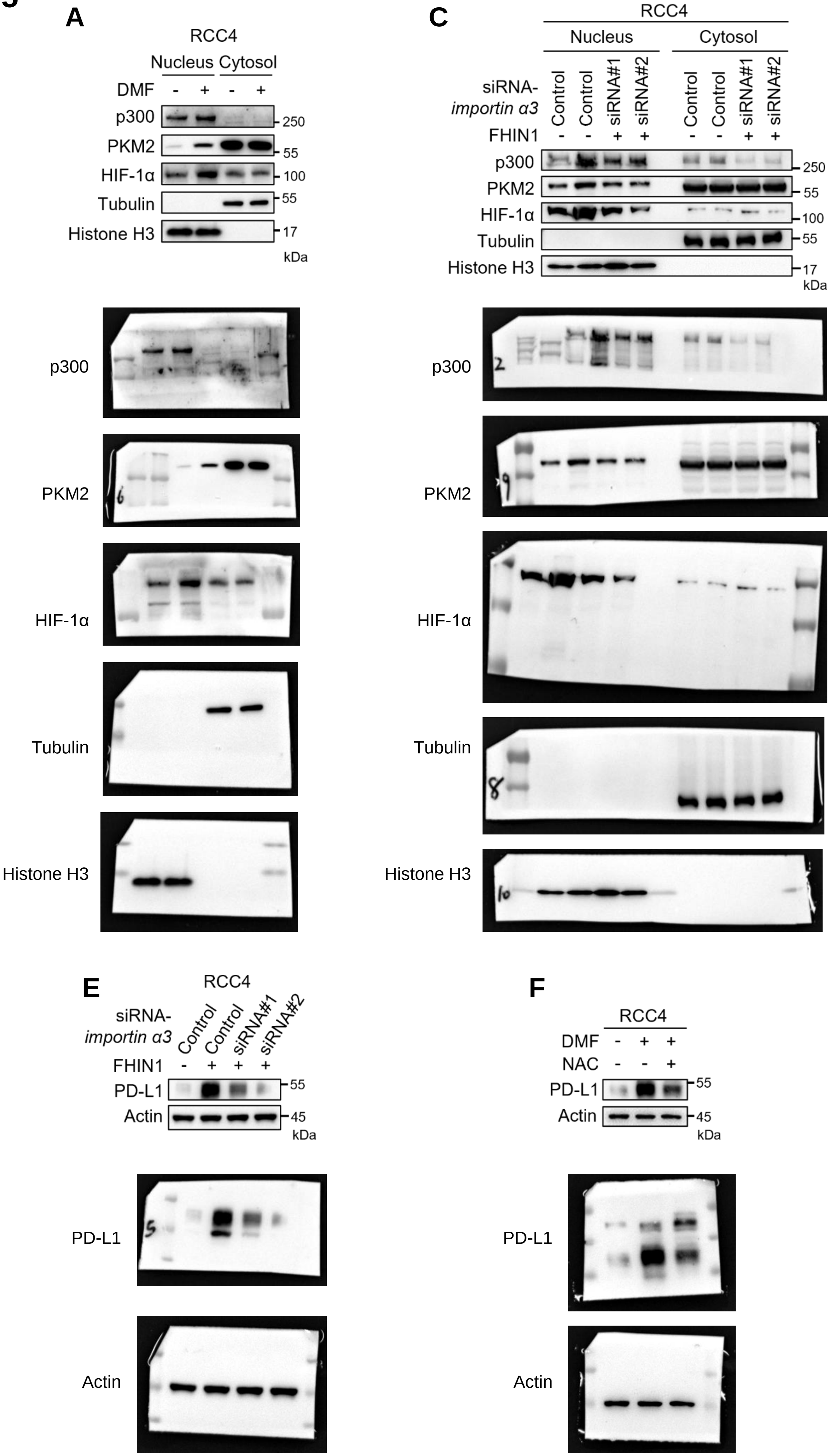

A
RCC4
Nucleus Cytosol
DMF - + - +
p300
PKM2
HIF-1α
Tubulin
Histone H3
250
55
100
55
17
kDa
C
RCC4
Nucleus
Cytosol
siRNA-
importin α3
Control Control siRNA#1 siRNA#2 Control Control siRNA#1 siRNA#2
FHIN1 - - + + - - + +
p300
PKM2
HIF-1α
Tubulin
Histone H3
250
55
100
55
17
kDa
E
RCC4
siRNA-
importin α3
Control Control siRNA#1 siRNA#2
FHIN1 - + + +
PD-L1
Actin
55
45
kDa
F
RCC4
DMF - + +
NAC - - +
PD-L1
Actin
55
45
kDa

Figure 5

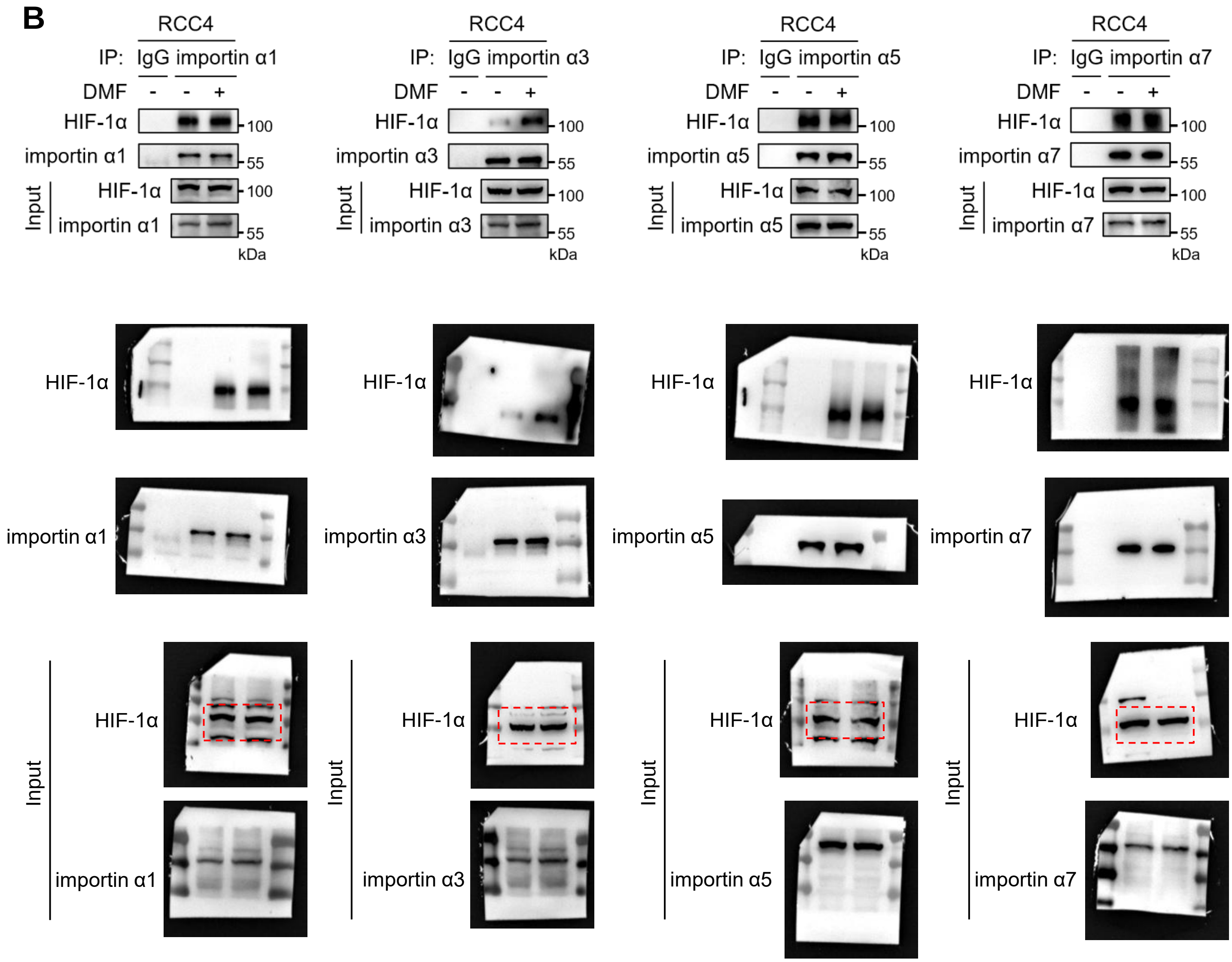
B
RCC4
IP: IgG importin α1
DMF - - +
HIF-1α
importin α1
Input
100
55
kDa
RCC4
IP: IgG importin α3
importin α3
RCC4
IP: IgG importin α5
importin α5
RCC4
IP: IgG importin α7
importin α7

Figure 5

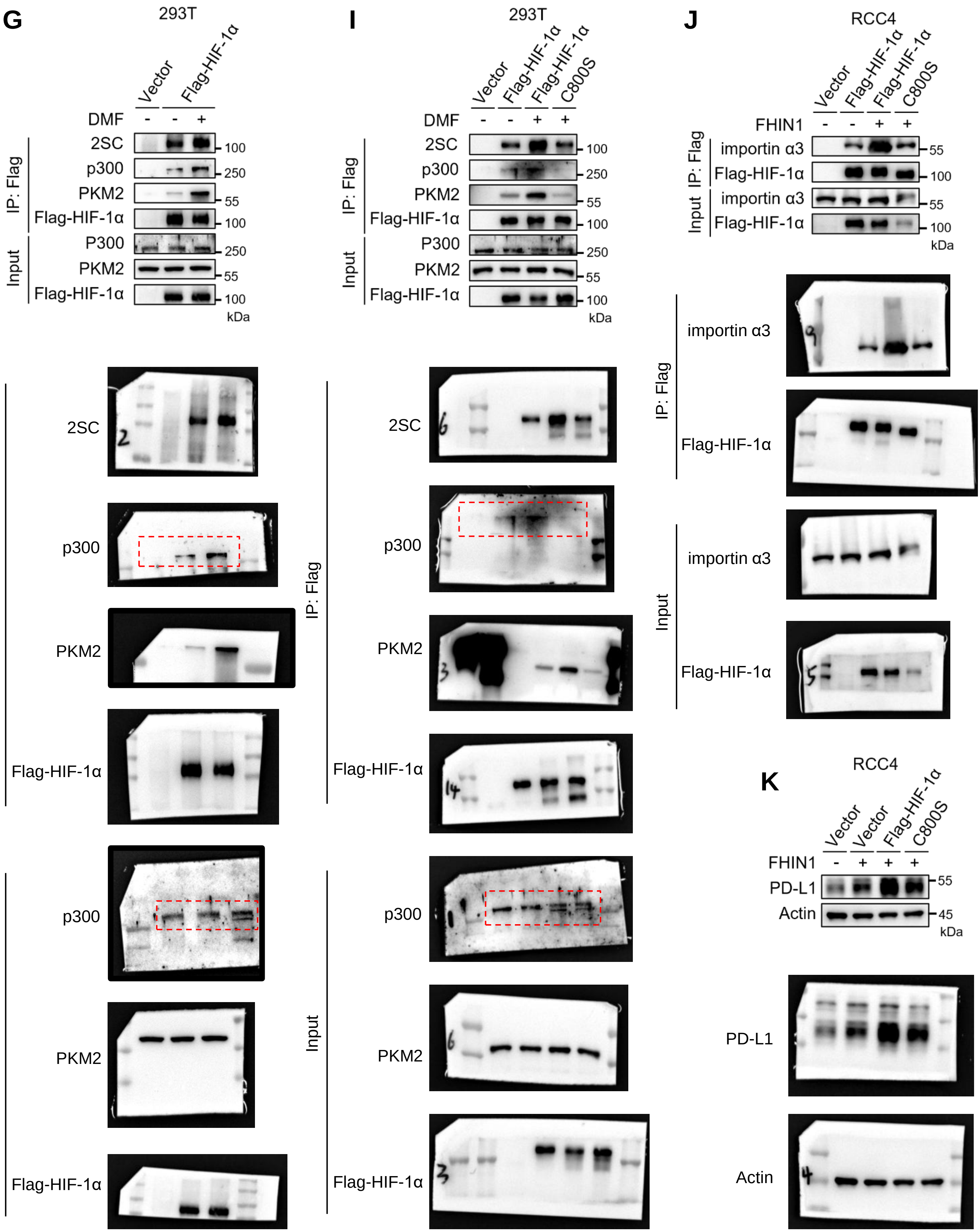
G
293T
Vector
Flag-HIF-1α
DMF - - +
IP: Flag
2SC
p300
PKM2
Flag-HIF-1α
Input
P300
PKM2
Flag-HIF-1α
100
250
55
100
250
55
100
kDa
I
293T
Vector
Flag-HIF-1α
Flag-HIF-1α C800S
DMF - - + +
IP: Flag
2SC
p300
PKM2
Flag-HIF-1α
Input
P300
PKM2
Flag-HIF-1α
100
250
55
100
250
55
100
kDa
J
RCC4
Vector
Flag-HIF-1α
Flag-HIF-1α C800S
FHIN1 - - + +
IP: Flag
importin α3
Flag-HIF-1α
Input
importin α3
Flag-HIF-1α
55
100
55
100
kDa
IP: Flag
importin α3
Flag-HIF-1α
Input
importin α3
Flag-HIF-1α
IP: Flag
2SC
p300
PKM2
Flag-HIF-1α
Input
p300
PKM2
Flag-HIF-1α
IP: Flag
2SC
p300
PKM2
Flag-HIF-1α
Input
p300
PKM2
Flag-HIF-1α
K
RCC4
Vector
Vector
Flag-HIF-1α C800S
FHIN1 - + + +
PD-L1
Actin
55
45
kDa
PD-L1
Actin

Supplemental Figure 1

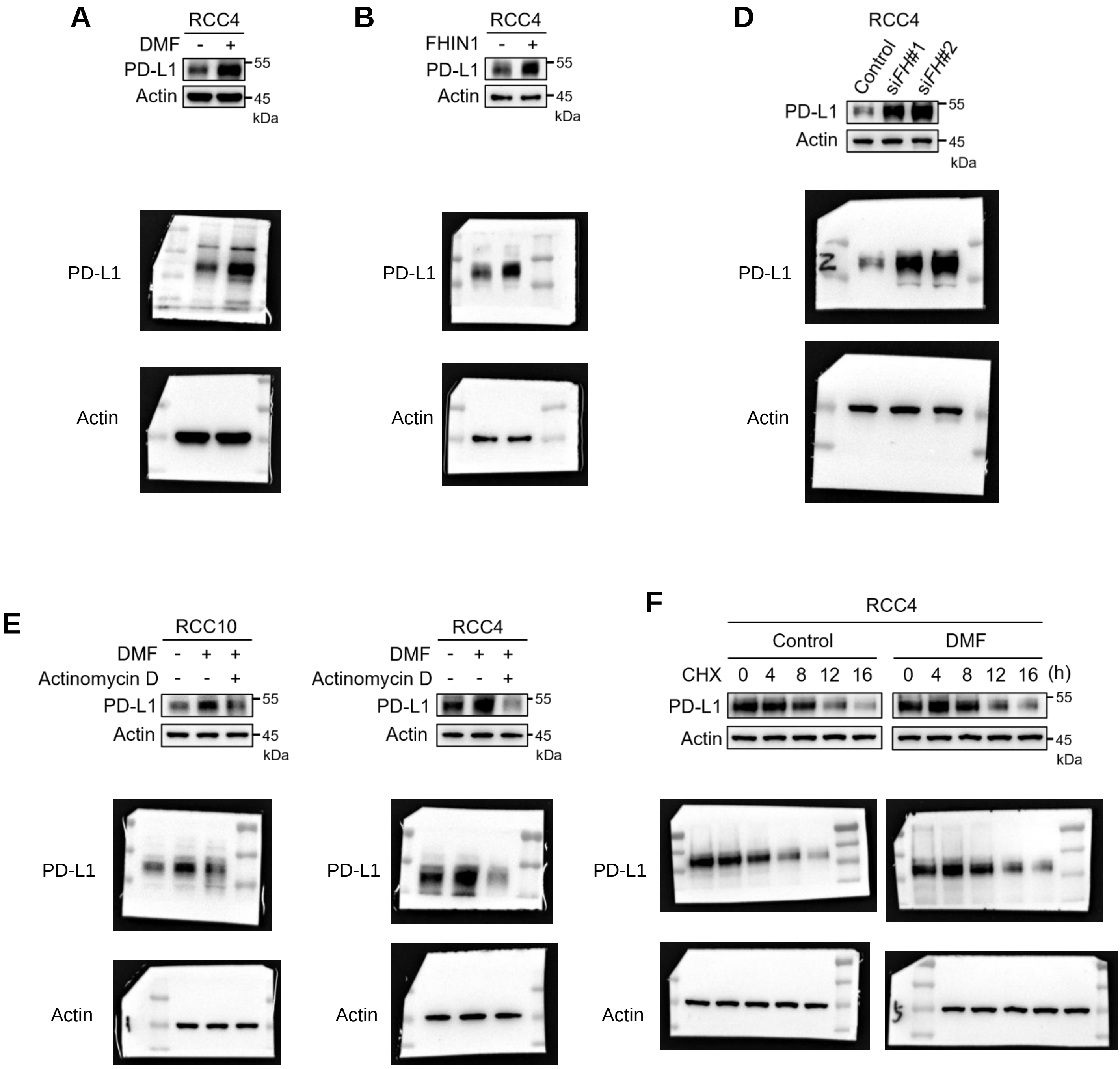

Supplemental Figure 2

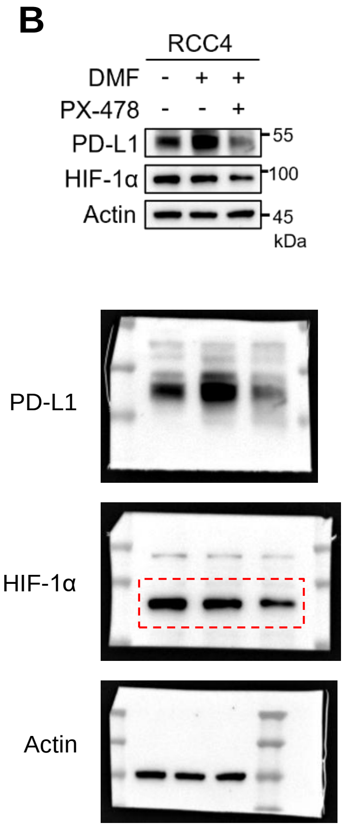

Supplemental Figure 3

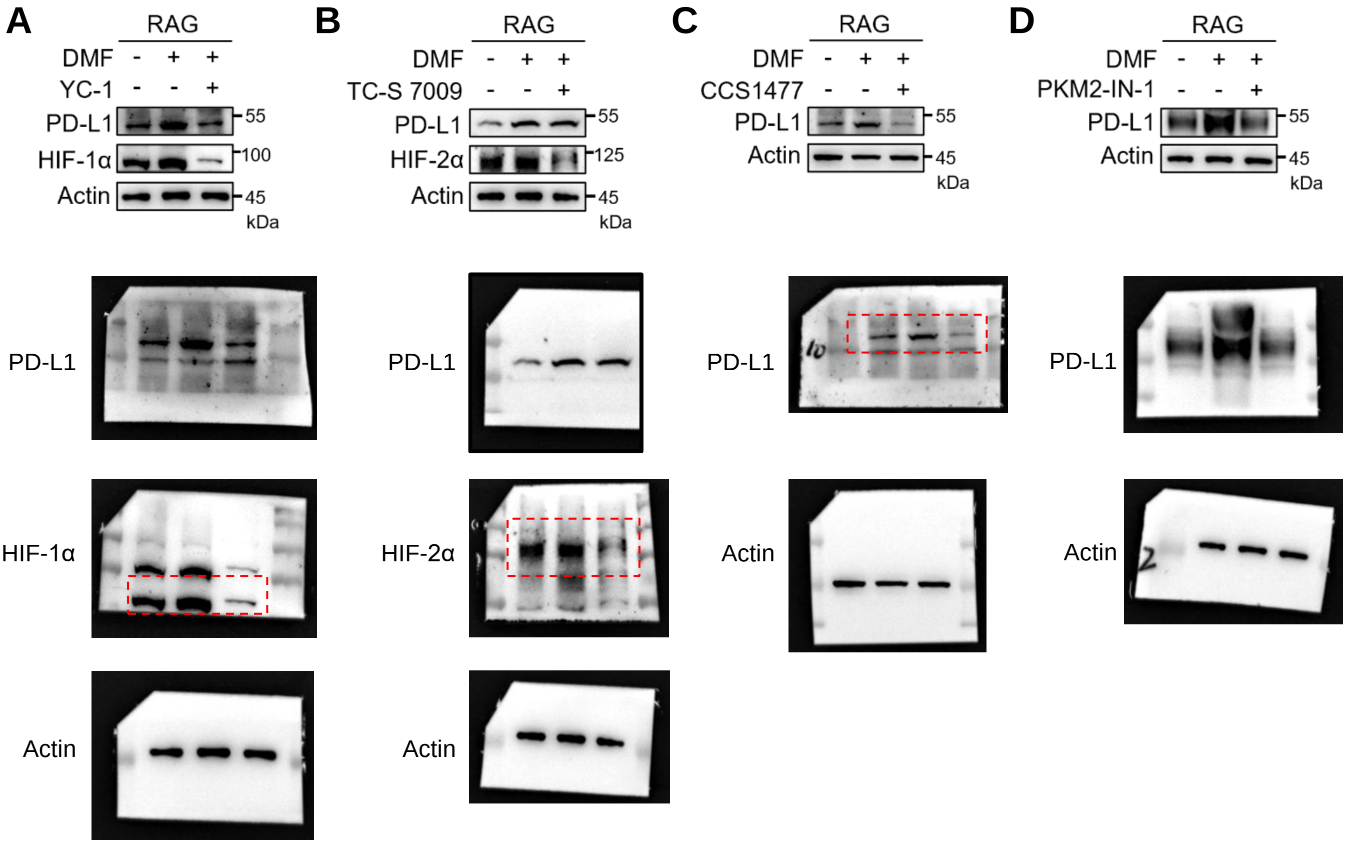

Supplement: Supplementary file 3 — Original Data Files [file 41419_2025_7752_MOESM3_ESM.pdf]
